# Cryptic circulation of chikungunya virus in São Jose do Rio Preto, Brazil, 2015–2019

**DOI:** 10.1371/journal.pntd.0012013

**Published:** 2024-03-14

**Authors:** Nathalia Zini, Matheus Henrique Tavares Ávila, Natalia Morbi Cezarotti, Maisa Carla Pereira Parra, Cecília Artico Banho, Livia Sacchetto, Andreia Francesli Negri, Emerson Araújo, Cintia Bittar, Bruno Henrique Gonçalves de Aguiar Milhin, Victor Miranda Hernandes, Karina Rocha Dutra, Leonardo Agopian Trigo, Leonardo Cecílio da Rocha, Rafael Alves da Silva, Gislaine Celestino Dutra da Silva, Tamires Fernanda Pereira dos Santos, Beatriz de Carvalho Marques, Andresa Lopes dos Santos, Marcos Tayar Augusto, Natalia Franco Bueno Mistrão, Milene Rocha Ribeiro, Tauyne Menegaldo Pinheiro, Thayza Maria Izabel Lopes dos Santos, Clarita Maria Secco Avilla, Victoria Bernardi, Caroline Freitas, Flora de Andrade Gandolfi, Hélio Correa Ferraz Júnior, Gabriela Camilotti Perim, Mirella Cezare Gomes, Pedro Henrique Carrilho Garcia, Rodrigo Sborghi Rocha, Tayna Manfrin Galvão, Eliane Aparecida Fávaro, Samuel Noah Scamardi, Karen Sanmartin Rogovski, Renan Luiz Peixoto, Luiza Benfatti, Leonardo Teixeira Cruz, Paula Patricia de Freitas Chama, Mânlio Tasso Oliveira, Aripuanã Sakurada Aranha Watanabe, Ana Carolina Bernardes Terzian, Alice de Freitas Versiani, Margareth Regina Dibo, Francisco Chiaravalotti-Neto, Scott Cameron Weaver, Cassia Fernanda Estofolete, Nikos Vasilakis, Mauricio Lacerda Nogueira

**Affiliations:** 1 Laboratório de Pesquisas em Virologia, Departamento de Doenças Dermatológicas, Infecciosas e Parasitárias, Faculdade de Medicina de São José do Rio Preto, São José do Rio Preto, São Paulo, Brazil; 2 Vigilância Epidemiológica, Secretaria de Saúde de São José do Rio Preto, São José do Rio Preto, São Paulo, Brazil; 3 Department of Strategic Coordination of Health Surveillance, Secretary of Health Surveillance, Brazilian Ministry of Health, Rio de Janeiro, Brazil; 4 Laboratório de Estudos Genômicos, Instituto de Biociências, Letras & Ciências Exatas, Universidade Estadual Paulista, São José do Rio Preto, São Paulo, Brazil; 5 Laboratório de Investigação de Microrganismos, Departamento de Doenças Dermatológicas, Infecciosas e Parasitárias, Faculdade de Medicina de São José do Rio Preto, São José do Rio Preto, São Paulo, Brazil; 6 Universidade Federal da Grande Dourados, Dourados, Mato Grosso do Sul, Brazil; 7 Centro Integrado de Pesquisa Hospital de Base, FUNFARME, São José do Rio Preto, São Paulo, Brazil; 8 Laboratório de Retrovirologia, Departamento de Medicina, Universidade de São Paulo, São Paulo, Brazil; 9 Instituto de Ciências Biológicas, Departamento de Parasitologia e Microbiologia, Universidade Federal de Juiz de Fora, Juiz de Fora, Minas Gerais, Brazil; 10 Laboratório de Imunologia Celular e Molecular, Instituto René Rachou, Fundação Osvaldo Cruz, Belo Horizonte, Minas Gerais, Brazil; 11 Department of Pathology, University of Texas Medical Branch, Galveston, Texas, United States of America; 12 Laboratório de Entomologia, Superintendência de Controle de Endemias, São Paulo, Brazil; 13 Departamento de Epidemiologia, Faculdade de Saúde Pública, Universidade de São Paulo, São Paulo, Brazil; 14 Department of Microbiology & Immunology, University of Texas Medical Branch, Galveston, Texas, United States of America; 15 Center for Biodefense and Emerging Infectious Diseases, University of Texas Medical Branch, Galveston, Texas, United States of America; 16 Center for Tropical Diseases, University of Texas Medical Branch, Galveston, Texas, United States of America; 17 Institute for Human Infections and Immunity, University of Texas Medical Branch, Galveston, Texas, United States of America; 18 Hospital de Base, FUNFARME, São José Do Rio Preto, São Paulo, Brazil; 19 Center for Vector-Borne and Zoonotic Diseases, University of Texas Medical Branch, Galveston, Texas, United States of America; Australian Red Cross Lifelood, AUSTRALIA

## Abstract

**Background:**

Chikungunya virus (CHIKV) has spread across Brazil with varying incidence rates depending on the affected areas. Due to cocirculation of arboviruses and overlapping disease symptoms, CHIKV infection may be underdiagnosed. To understand the lack of CHIKV epidemics in São José do Rio Preto (SJdRP), São Paulo (SP), Brazil, we evaluated viral circulation by investigating anti-CHIKV IgG seroconversion in a prospective study of asymptomatic individuals and detecting anti-CHIKV IgM in individuals suspected of dengue infection, as well as CHIKV presence in *Aedes* mosquitoes. The opportunity to assess two different groups (symptomatic and asymptomatic) exposed at the same geographic region aimed to broaden the possibility of identifying the viral circulation, which had been previously considered absent.

**Methodology/principal findings:**

Based on a prospective population study model and demographic characteristics (sex and age), we analyzed the anti-CHIKV IgG seroconversion rate in 341 subjects by ELISA over four years. The seroprevalence increased from 0.35% in the first year to 2.3% after 3 years of follow-up. Additionally, we investigated 497 samples from a blood panel collected from dengue-suspected individuals during the 2019 dengue outbreak in SJdRP. In total, 4.4% were positive for anti-CHIKV IgM, and 8.6% were positive for IgG. To exclude alphavirus cross-reactivity, we evaluated the presence of anti-Mayaro virus (MAYV) IgG by ELISA, and the positivity rate was 0.3% in the population study and 0.8% in the blood panel samples. In CHIKV and MAYV plaque reduction neutralization tests (PRNTs), the positivity rate for CHIKV-neutralizing antibodies in these ELISA-positive samples was 46.7%, while no MAYV-neutralizing antibodies were detected. Genomic sequencing and phylogenetic analysis revealed CHIKV genotype ECSA in São José do Rio Preto, SP. Finally, mosquitoes collected to complement human surveillance revealed CHIKV positivity of 2.76% of *A*. *aegypti* and 9.09% of *A*. *albopictus* (although it was far less abundant than *A*. *aegypti*) by RT–qPCR.

**Conclusions/significance:**

Our data suggest cryptic CHIKV circulation in SJdRP detected by continual active surveillance. These low levels, but increasing, of viral circulation highlight the possibility of CHIKV outbreaks, as there is a large naïve population. Improved knowledge of the epidemiological situation might aid in outbreaks prevention.

## Author summary

Chikungunya virus (CHIKV) circulation has been causing large epidemics throughout the tropics and subtropics, affecting the quality of life of the global population. Additionally, the asymptomatic or subclinical case numbers are unclear due to a paucity of epidemiological studies. Since 2013, CHIKV epidemics have been spreading in the Americas, and more than 900,000 CHIKV infection cases were confirmed in Brazil in 2020. In São José do Rio Preto (SJdRP), only 41 cases of CHIKV infection were confirmed from 2015–2020, according to official bulletins. In the same period, our study revealed seven participants among 340 (2.05%) who seroconverted to anti-CHIKV IgG, while in a blood panel collected during a dengue outbreak, 22 samples among 497 (4.42%) had anti-CHIKV IgM. These results demonstrate that the cryptic CHIKV circulation in SJdRP and the high percentage of naive individuals could represent an imminent risk of outbreaks. Additionally, our data might assist public health authorities in the development of health measures for epidemiological and vector surveillance programs.

## Introduction

Chikungunya virus (CHIKV) is an arbovirus first isolated in Tanzania in 1952. Four lineages of CHIKV have been identified: West African (WA), East-Central-South African (ECSA), Asian, and Indian Ocean Lineage (IOL) [1]. All these lineages have the mosquitoes *Aedes aegypti*, and the IOL also has *A*. *albopictus* as primary vectors [2]. Since its discovery, CHIKV has been associated with sporadic outbreaks, especially in the Asian and African continents; in 2004, a new strain, IOL, emerged from the ECSA lineage in Kenya, resulting in large outbreaks with higher infectivity rates [reviewed by Weaver and Forrester [1,3]]. From Kenya, CHIKV spread to South and Southeast Asia and subsequently throughout much of the tropics and subtropics, causing epidemics in several countries [1,4]. In 2013, the first cases of autochthonous transmission of CHIKV were reported in the Americas, where the virus quickly disseminated to most countries in Latin America, causing a large epidemic [5].

In Brazil, CHIKV was detected first in 2014 in Amapá and Bahia states, and these outbreaks were associated with the Asian and ECSA genotypes, respectively [6]. After that, the virus caused further outbreaks in several states, predominantly in the northeastern and southeastern regions, resulting in localized, extensive epidemics and more than 800,000 cases since its emergence [7,8]. Currently, CHIKV cocirculates with other arboviruses of clinical relevance and is the causative agent of a febrile syndrome, which presents with symptoms overlapping those of other arboviral infections, such as those caused by dengue (DENV), Zika (ZIKV), yellow fever (YFV), and Mayaro viruses (MAYV) [9–13]. Moreover, owing to the substantial number of annual dengue cases and the overlapping symptoms among arboviral infections, many viral infections can be underdiagnosed or misdiagnosed as dengue. Cases of chikungunya fever (CHIKF) that are not precisely reported make it difficult for health authorities to monitor increases in the number of CHIKF cases in the population and compromise epidemiological surveillance as part of disease prevention and control measures.

CHIKV infection may be clinically characterized as an acute febrile disease markedly associated with joint involvement ranging from mild arthralgia to intense and debilitating polyarthritis lasting for months or years [4,14]. Despite the importance of symptomatic cases, asymptomatic (unapparent or subclinical) infections have been described at a frequency of up to 25% [as reviewed by [15]]. Generally, such asymptomatic infections occur before and during CHIKF epidemics, impacting public health, as the number of individuals exposed to the virus may be unknown [16–18].

A systematic review of seroprevalence showed anti-CHIKV IgG positivity rates ranging from 0.2 to 88.6% worldwide [19]. In the Americas, the highest rates were observed in Martinique and Guadeloupe (48.1%), followed by Brazil (36.2%) and Mexico (29.5%) [19]. Notably, there were 457,568 confirmed cases in Brazil between 2017 and 2021. In 2017, most cases (36.35%) were reported in Ceará State, in the Northeast region, and Rio de Janeiro State, in the Southeast region, accounted for the most cases in the country in 2018 and 2019. Over these five years, São Paulo state reported 16,451 confirmed CHIKV infections (3.6%), 90.1% of which occurred in 2021 [7].

São José do Rio Preto (SJdRP), São Paulo State (SP), Brazil, is an important epidemiological site in the arboviral context and has reported cocirculation of arboviruses in recent years. Since 2015, the city has reported outbreaks of dengue detected by an active and well-established arboviral surveillance program. Despite the epidemic history, the city has not yet reported a CHIKF outbreak, although CHIKV has been circulating widely in Brazil. Between 2015 and 2020, only 41 cases of CHIKV infection (0.05%) were confirmed among 81,149 arboviral infection-confirmed cases in SJdRP [20,21]. Such divergence supported the need for this study to investigate CHIKV circulation in SJdRP. To investigate such differences and the gap in the distribution of CHIKF cases in SJdRP concerning other arboviruses, we performed two different but linked epidemiological studies (1 and 2) carried out over four years (2015–2019). During this period, we followed the circulation of CHIKV based on confirmed cases by investigating official bulletins, and we identified the previously exposed population by CHIKV IgG antibodies through ELISA and plaque-reduction neutralization tests (PRNT) to monitor virus circulation. We conducted entomological studies in the study area of study 1 to examine the increase in *Aedes* mosquito-driven CHIKV infection and anticipate future epidemics. Finally, we performed a phylogenetic analysis to verify the CHIKV lineage circulating in SJdRP.

The purpose of this study was to demonstrate that the emergence of epidemics caused by a new virus (etiological agent) can be monitored with an active surveillance program by integrating the work of multisectoral researchers with public health departments in the city, involving acute cases and differential diagnosis, prospective study of asymptomatic individuals, and vector studies. In this way, after the introduction of CHIKV, there is time to develop measures for epidemic control and prevention.

## Methods

### Ethics statement

This prospective study was approved by the Internal Review Board (IRB) of Faculdade de Medicina de São José do Rio Preto (FAMERP) (protocol number 32993014.0.0000.5415, approved on 09 September 2014). Signed consent forms were obtained from all individuals aged over 18 years who decided to participate. For those aged younger than 18 years old, their parents or legal guardians provided written consent for their participation in the study. The study of blood panel samples and waiver of consent (since these samples were collected for routine epidemiological surveillance by the public health authority) were approved by the Internal Review Board (IRB) of Faculdade de Medicina de São José do Rio Preto (FAMERP) (protocol number 02078812.8.0000.5415, approved on 03 July 2012).

### Study area

**Fig 1:**
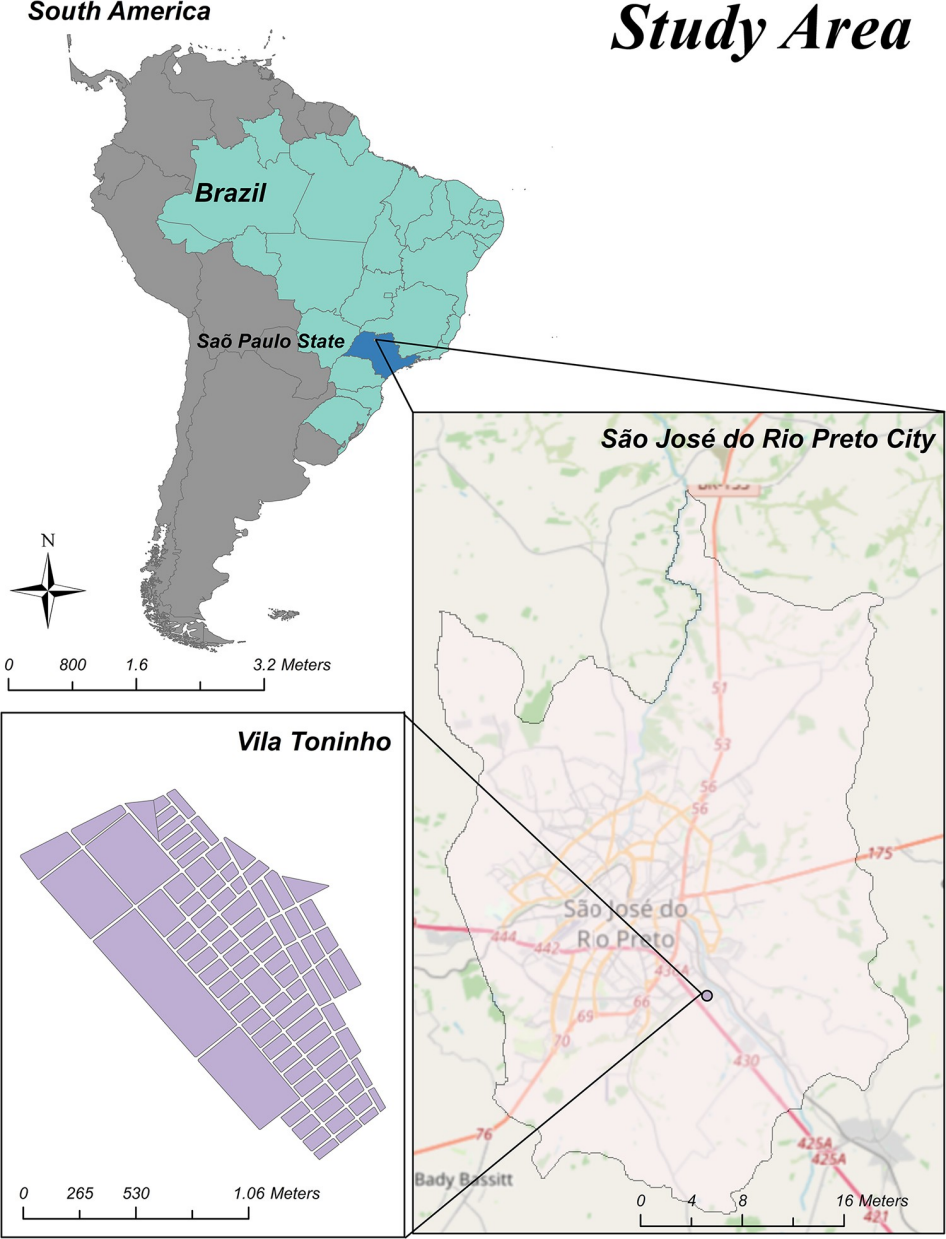
Area of Study. (A) Geopolitical map of South America, showing Brazil (dark green), and São Paulo state (with the latter highlighted in green, São José do Rio Preto (black point), (B) São José do Rio Preto located in the northeastern region of São Paulo State, and (C) the Vila Toninho neighborhood located in the Southeastern part of the city. Shapefile downloaded from https://portaldemapas.ibge.gov.br/portal.php#homepage.

SJdRP is located in the northwestern region of São Paulo state (20°48’36” S and 49°22’59” W), Brazil (Fig 1B). It has an estimated population of 469,173 inhabitants (48.02%, 225,296 male; and 51.98%, 243,877 female) and a Municipal Human Development Index (MHDI) of 0.797 [22]. The health system of SJRP comprises ten areas that cover the whole population of the city. Each site has a Basic Family Health Unit (BFHU) as a reference health center. As part of the established active surveillance system of SJRP, when the BFHU assistance team identifies arboviral infection-suspected cases, blood samples are collected from consented symptomatic patients and sent to the Laboratório de Pesquisas em Virologia (LPV) in the Faculdade de Medicina de São José do Rio Preto (FAMERP) to perform arboviral molecular investigation.

### Population study

This study was performed based on two studies: i) the first was composed of residents of the Vila Toninho neighborhood (Fig 1C), and they were asymptomatic participants from the prospective study for arboviral surveillance between 2015 and 2019; ii) the other one was composed of dengue-suspected cases from the SJdRP general population during the 2019 outbreak, from whose blood samples were collected when they were symptomatic when they were seen in the health care units, representing differential diagnosis of arboviral infections. The choice of study design aimed demonstrating the cryptic CHIKV circulation over the years by study 1, and in addition, with study 2, the misdiagnosed cases of Chikungunya infection during a dengue outbreak, where the possibility of underdiagnosis is high, especially for symptoms overlapping of both diseases.

### Study 1—Prospective surveillance study in the Vila Toninho neighborhood

The Vila Toninho neighborhood is located in the southeastern region of SJdRP, with 11,429 inhabitants, including 5,923 (51.82%) females and 5,506 (48,18%) males in 2015 [22]. Most of its population is economically active and ranges from 14 to 60 years old. In 2015, the first year of this study, the neighborhood was composed of 5,911 households, with an occupancy rate of 93% [22].

To understand the circulation of CHIKV in this population, this study was performed via face-to-face interviews and blood collection for four years, from enrollment to the last annual follow-up. The recruitment of the individuals occurred from October 2015 to March 2016, referred to as the follow-up baseline (FB) 2015–2016. In the first-year follow-up (A01), the visits were carried out from October 2016 to March 2017; in the second-year follow-up (A02), from October 2017 to March 2018; and in the third-year follow-up (A03), from October 2018 to March 2019.

The inclusion criteria for enrollment in this study were as follows: 1) agreed to participate and signed the consent form, 2) answered the sociodemographic questionnaire and provided a sample of peripheral blood, 3) were ten years old or older, and 4) were a resident of the Vila Toninho neighborhood. The exclusion criteria were 1) refusal to sign the consent form and 2) age less than ten years.

On admission to the study, all participants answered a sociodemographic questionnaire and had their blood samples collected to determine prior exposure to CHIKV through serological tests. At all follow-up visits (A01, A02, and A03), performed annually, new blood samples from the participants were collected, and the sociodemographic data were updated through standard questionnaires, in which answers were self-reported. All blood samples were collected and sent to the LPV, where they were stored at -80°C until analysis.

### Study 2—Panel of blood samples from dengue-suspected symptomatic individuals

Dengue-suspected symptomatic individuals in SJdRP, when undergoing clinical evaluation in some health care units, had blood samples collected as recommended by the Public Health Authority for arbovirus investigation. Such samples were already routinely sent to the LPV, where molecular and serological tests are performed for dengue diagnosis and differential diagnosis for CHIKV or ZIKV infections. To achieve the study objectives, blood samples collected after seven days from dengue-like symptom onset during the 2019 dengue outbreak in SJdRP were considered eligible for CHIKV investigation and included in this study when anti-dengue and anti-Zika IgM were not detected. Demographic and clinical data from such individuals were obtained from SINAN (Sistema de Informação de Agravos de Notificação) forms, which were reported when the patients were supported in the health care unit.

### Laboratory investigation

#### Enzyme-linked immunosorbent assay (ELISA)

To investigate the seroconversion for CHIKV in the study from the Vila Toninho neighborhood over the study period, the longitudinal blood samples collected annually from baseline until follow-up A03 were paired and subjected to anti-CHIKV IgG detection using an ELISA kit (EUROIMMUN, Lübeck, Germany-GE). All participants were asked about the presence of symptoms compatible with arboviral infection over the last follow-up years. Due to the possibility of cross-reactivity between alphaviruses [23], we tested all anti-CHIKV IgG-positive or borderline-positive samples for MAYV IgG using an anti-MAYV IgG ELISA kit (EUROIMMUN, Lübeck, Germany-GE). All analyses were performed following the manufacturer’s instructions (www.euroimmun.com.br), and the plates were read at 450 nm using an ELISA reader (Molecular Devices, LLC, San Jose, CA, U.S.A).

#### Plaque reduction neutralization test (PRNT)

When longitudinal blood samples were positive for anti-CHIKV IgG and/or anti-MAYV IgG antibodies in the previously described steps, they were submitted to a PRNT to exclude the possibility of cross-reactivity between the two alphaviruses. To this end, CHIKV and MAYV stocks were maintained in C6/36 cells in Leibovitz-15 medium (L-15, Cultilab, Brazil) at 28°C. The media were supplemented with 1% fetal bovine serum (FBS) (Cultilab, Brazil), 100 U/mL penicillin, and 100 μg/mL streptomycin (GIBCO, U.S.A.) every two days, as previously described [24]. The CHIKV and MAYV supernatants were collected, aliquoted, and identified, and viral stocks were stored at -80°C. Next, they were tittered by plaque assay in Vero cells to perform the PRNT.

The virus strains employed in the assay included a CHIKV isolated from a patient in Pernambuco state, Brazil, and MAYV strain BeAr20290 (provided by Dr. Pedro Vasconcelos) [25]. Serum samples underwent inactivation at 56°C for 60 minutes and were then subjected analysis to determine specific neutralization antibody titers, following previously described methods [26–28].

Briefly, viral dilutions (approximately 50 plaque-forming units) were initially paired with screening serum dilutions (1:10) to identify samples exhibiting negative or positive for CHIKV and MAYV neutralization activity. The serum/virus mixture was removed and subsequently incubated at 37°C in a humidified atmosphere with 5% CO2 for one hour. Next, PRNT was performed in 24-well plates containing Vero cells, and the mixture was added and incubated for one hour for adsorption. Finally, the serum/virus mixture was removed, and added semisolid medium to the cell monolayer until fixation with cold paraformaldehyde 4% and stained with crystal violet. The results were expressed as plaque-forming units/mL (PFU/mL).

The samples submitted to the screening considered positive were those with neutralizing antibodies titers >1:10. So, the positive samples, ranging from 1:20 to 1:2,560, were submitted to PRNT with serial dilutions for verify CHIKV and MAYV neutralizing antibodies titers. Samples with a PRNT_80_<10 during screening were considered negative. The plates were stained with crystal violet, and the results were expressed as PFU/mL. A negative sample for CHIKV, DENV, and ZIKV (confirmed by ELISA and PRNT) was used as a validation control in the PRNT assay.

#### Molecular investigation and sequencing

To identify the circulating CHIKV genotype in the region, we subjected samples from a panel of dengue**-**suspected patients during the 2019 outbreak for cDNA synthesis followed by one-step real-time polymerase chain reaction (RT–qPCR) according to reference [29]. The CHIKV RT–PCR-positive samples were tested with end-point PCR, as described by reference [30], using six pairs of primers targeting structural genes (E2, 6K, and E1) of the CHIKV genome (S1 Table). The amplified fragments (~1,170 bp) were purified and sequenced using a BigDye Terminator Cycle Sequencing Read Reaction Kit v3.1 (Foster City, CA, U.S.A.) in an ABI3130 automatic sequencer (U.S.A.) using the same set of primers cited below and described in S1 Table, when Ct values allowed.

#### Phylogenetic analysis

The consensus sequences were generated using Geneious Prime 2023.0 (https://www.geneious.com) and aligned with 172 CHIKV sequences (S2 Table) deposited at GenBank (https://www.ncbi.nlm.nih.gov/genbank/) using MAFFT multiple sequence alignment software version 7.271 [31]. A phylogenetic tree was reconstructed using the Maximum-likelihood (ML) method in IQ-TREE v.2.0.3 [32], using the TIM2e+G4 as the best-fit model of nucleotide substitution according to Bayesian information criterion (BIC) inferred by ModelFinder [33]. The reliability of branching patterns was tested using a combination of Ultrafast Bootstrap (UFBoot, 1,000 alignments) and SH-like approximate likelihood ratio test (SH-aLRT, 1,000 replicates). The final tree was visualized and edited in iTOL version 6.6 [34].

#### Mosquito sampling, processing, and molecular investigation

To corroborate the CHIKV circulation in the study from the Vila Toninho neighborhood, mosquitoes were collected once per month (in the last week of each month) from October 2015 to August 2019 in that area [35]. Then, the mosquitoes were sent to the LPV to be identified [36,37] and separated in pools, by species, sex, local and date of collection, with no more than 10 specimens each pool, and tested by molecular methods for virus presence. Total RNA extraction and RT–qPCR were performed according to Machado et al. [38] and Lanciotti et al [29].

#### Spatial distribution database

Data generated by analyses of mosquito and human samples of the two studies were used to create maps with the spatial distribution of CHIKV infection cases. Maps were generated using TerraView software (INPE) version 4.2.2 and ArcGIS software (ESRI) version 10.2.2, and thematic maps were built in ArcMap (version 10.8.1).

#### Rates and statistical analyses

**Fig 2:**
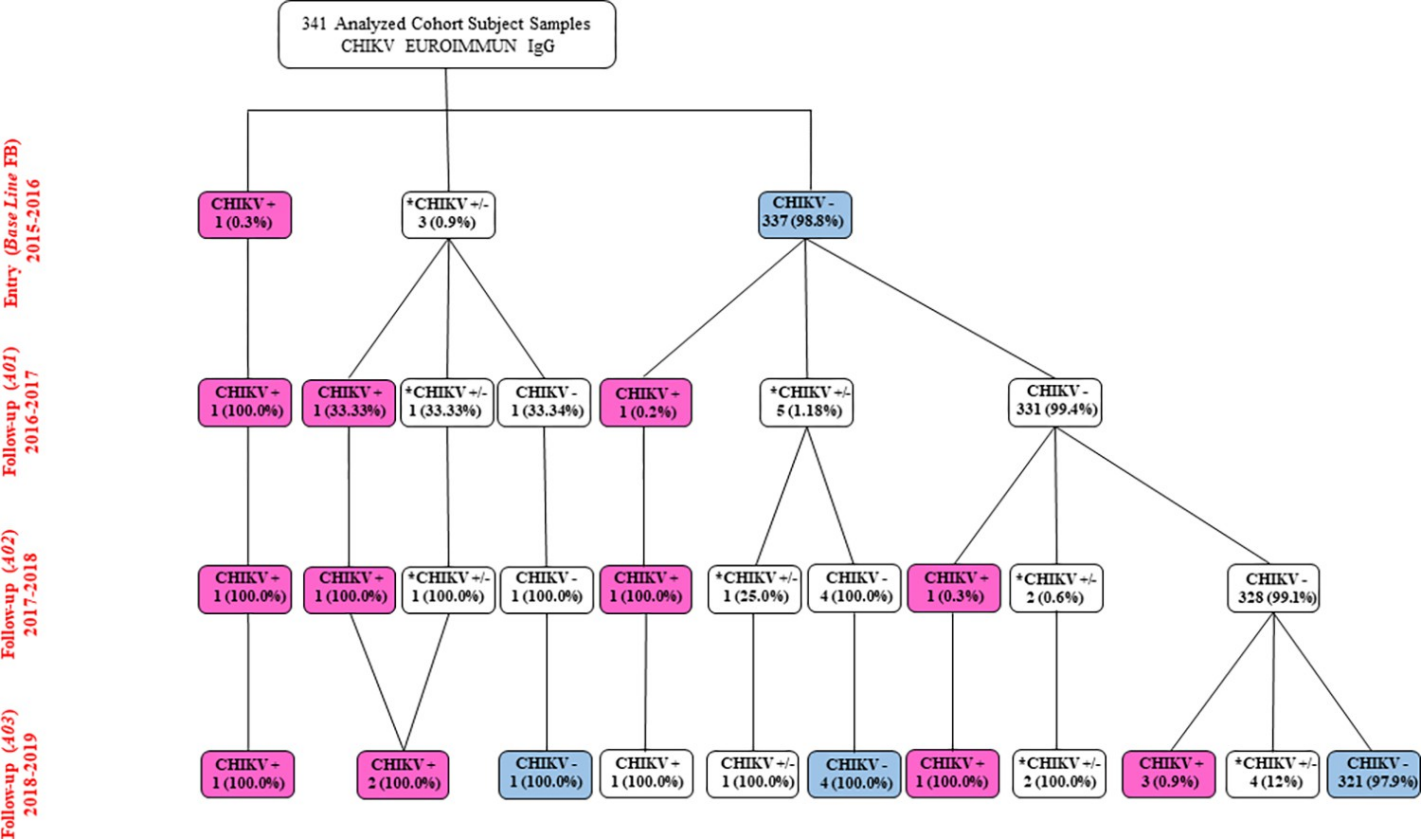
CHIKV antibodies of participants in the study in the Vila Toninho neighborhood. **Subtitle:** Flowchart showing the presence of chikungunya antibodies (Anti-CHIKV IgG) in participants in the study over four years, during the collection period that occurred between October and March of 2015–2019 (Entry Baseline 2015/2016, Follow-up A01 2016/2017, Follow-up A02 2017/2018 and Follow-up A03 2018–2019, as demonstrated in each line of the flowchart) in the Vila Toninho neighborhood. The participants presenting chikungunya-positive antibodies (CHIKV +) are highlighted in pink, while those without chikungunya antibodies (CHIKV -) are shown in blue. CHIKV +: positive samples; *CHIKV +/-: equivocal samples; and CHIKV -: negative samples, as determined by anti-CHIKV IgG ELISA.

We calculated the number of new cases in a period according to the risk of population exposure in the same period and expressed it as a percentage. The CHIKF incidence rate in the study period of the study from the Vila Toninho neighborhood was calculated through the ratio between the number of anti-CHIKV IgG-positive individuals in A03 and the number of individuals naïve to CHIKV in follow-up baseline (x 1,000 inhabitants), as referred (Fig 2). The infection rate was calculated through the ratio between all anti-CHIKV IgG-positive individuals from FB to A03 and the total number of patients in the study (%), both yearly and over three years. For the statistical analyses, the variables were grouped as follows: (*i*) sex (male and female) and (*ii*) four age ranges (10–20, 21–40, 41–60, and ≥ 61 years). First, continuous variables were tested for normality by the Kolmogorov–Smirnov method. The nonparametric Kruskal–Wallis test with Dunn’s correction and the Mann–Whitney U test were used to compare differences between means using GraphPad Prisma software (version 8.0.1). The symptom profile presented in group 2 was created in Epi Info Launch TM 7.2.2.6 software. The characteristics were considered statistically significant when a two-tailed p-value was <0.05 and the confidence interval was 95%. Significance was considered when a confidence interval of 95% was impossible to achieve due to the sample size.

## Results

### Surveillance of arboviruses in São José do Rio Preto

To understand CHIKV distribution and possible large epidemics in São José do Rio Preto, we followed the level of virus circulation according to the cases officially reported by Vigilância Epidemiológica de SJdRP. Between 2015 and 2020, the municipal surveillance system reported a total of 118,667 dengue-, Zika- or chikungunya-suspected cases in SJdRP, in accordance with the epidemiologic bulletins [39]. Among them, 81,149 (68.4%) were confirmed by molecular tests, with 0.05% (41/81,149) CHIKF and 95.95% (81,108/81,149) caused by other arboviruses, i.e., DENV and ZIKV (Table 1).

**Table 1:** Chikungunya-confirmed cases in São José do Rio Preto (SJRP), SP, between 2015 and 2019, according with epidemiological bulletins.

	Chikungunya		Dengue and Zika	
Year	Notified	Confirmed	Notified	Confirmed
2015	2	2	28,315	22,234
2016	96	6	27,155	16,608
2017	80	14	3,369	637
2018	100	12	4,216	1,219
2019	117	5	43,481	33,158
2020	37	2	11,668	7,252
Total	432	41	118,204	81,108

To identify the origin of infection, we differentiated between autochthonous and imported CHIKV cases. Among the 41 CHIKV-confirmed cases, 73.2% (n = 30) were autochthonous, and 26.8% (n = 11) were probably imported, according to governmental bulletins [40]. In addition, 9.8% (4/41) involved individuals living in the Vila Toninho neighborhood, the same area where our prospective surveillance study and entomological studies were conducted. However, none of the CHIKV-confirmed cases were enrolled in our study. A low incidence of CHIKF was found in SJdRP despite local circulation, demonstrating that the cryptic circulation of CHIKV should be monitored since most of the naive population presents an imminent risk of an epidemic.

### Vila Toninho study

After CHIKV was confirmed in SJdRP, we started the first epidemiological study to assess the possibility of a CHIKF epidemic, as well as the prevalence of previous exposure to CHIKV in the study population in Vila Toninho for four years (2015–2019). The baseline characteristics of the study are described in Chiaravalloti-Neto et al [41].

According to the eligibility criteria, 1,517 individuals were recruited in the baseline year (FB 2015/2016); however, only 22.5% (n = 341) of participants remained in the study until the third-year follow-up and provided all longitudinal blood samples. Therefore, these 341 individuals composed the final study for our analysis. Most participants analyzed were females (63.0%, n = 215/341); were aged from 41 to 60 years old (41.3%, n = 141/341); were white (58.1%, n = 198/341); and had from 3 to 7 years of schooling (47.2%, n = 161/341) (S3 Table).

#### IgG seroprevalence and seroconversion over the study period

A total of 341 participants in the Vila Toninho population study had blood samples collected annually. As the samples were paired, it was possible to follow the anti-CHIKV IgG seroprevalence in individuals by ELISA over the years. In the FB, the positivity of anti-CHIKV IgG was 0.2% (1/341; CI 95% 0.0–1.6) in the FB, followed by a slight but consistent increase in the follow-up years, 0.8% (3/341; CI 0.3–2.6) in A01, 1.17% (4/341; CI 95% 0.4–3.0) in A02, and 2.34% (8/341; CI 95% 1.1–4.6) in A03, according with Fig 2. All individuals IgG+ remained positive in subsequent years.

The flowchart (Fig 2) shows that during the recruitment year (FB), one participant (0.2%) was anti-CHIKV IgG positive (pink), and three participants exhibited indeterminate serologic results (equivocal +/-). A similar condition occurred in six samples in A01, four in A02, and seven in A03. Out of 98.8% (n = 337/341) of individuals who resulted in negativity for anti-CHIKV IgG in the FB, 96.7% (n = 326/337) remained uninfected (blue) until the last year’s follow-up, A03 (Fig 2). The serological status detected in the study population demonstrates that the cryptic circulation of CHIKV in study 1 is similar to that in the municipality of SJdRP. The sociodemographic characteristics of the participants were not associated with positive serological IgG anti-CHIKV test results (Table 2).

**Table 2:** Anti-CHIKV IgG by ELISA Seroprevalence in the Population Study between 2015 and 2019 According to Age and Sex.

Demographic variable		Entry (Baseline FB) 2015/2016			Follow-up A01 2016/2017		Follow-up A02 2017/2018		Follow-up A03 2018/2019		Incidence	Infection
		Participants n (%)	Anti-CHIKV IgG (+) n (%)	95% CI	Anti-CHIKV IgG (+) n (%)	95% CI	Anti-CHIKV IgG (+) n (%)	95% CI	Anti-CHIKV IgG (+) n (%)	95% CI	1.000/hab.	rate (%)
	TOTAL	341 (100.0)	1 (0.3)	(0.0–1.6)	3 (0.9)	(0.3–2.6)	4 (1.2)	(0.4–3.0)	8 (2.3)	(1.1–4.6)	20.6	2.1
SEX												
	Male	126 (37.0)	1 (0.8)	(0.0–4.4)	2 (1.6)	(0.2–5.7)	2 (0.9)	(0.1–3.3)	4 (1.8)	(0.5–4.7)	24.6	0.9
	Female	215 (63.0)	0 (0.0)	(0.0)	1 (0.5)	(0.0–2.6)	2 (1.6)	(0.2–5.7)	4 (3.2)	(0.8–8.0)	18.6	1.2
AGE (years)												
	10–20	17 (5.0)	0 (0.0)	(0.0)	1 (5.9)	(0.1–28.7)	1 (5.9)	(0.15–28.7)	1 (5.9)	(0.1–28.7)	58.8	0.3
	21–40	65 (19.0)	1 (1.5)	(0.0–8.3)	1 (1.5)	(0.0–8.3)	2 (3.1)	(0.4–10.7)	3 (4.6)	(0.9–12.9)	31.5	0.9
	41–60	141 (41.3)	0 (0.0)	(0.0)	1 (0.7)	(0.02–3.9)	1 (0.7)	(0.02–3.9)	3 (2.1)	(0.4–6.1)	21.3	0.9
	> 61	118 (34.6)	0 (0.0)	(0.0)	0 (0.0)	(0.0)	0 (0.0)	(0.0)	1 (0.9)	(0.0–4.6)	8.5	0.3

Another relevant data point was the CHIKV infection rate. Since the first year, seven individuals seroconverted for anti-CHIKV IgG among 340 CHIKV-naive participants (anti-CHIKV IgG negative in the study baseline), resulting in an inferred infection rate of 2.05%, in addition to 0.6%, 0.3%, and 1.2% for A01, A02, and A03, respectively. The total CHIKV infection rate during the study was 2.1%, and the global incidence rate of CHIKF cases was 20.6 cases/1,000 inhabitants (Table 2). Our results demonstrated that CHIKV was introduced and maintained under low circulation in our study population in the SJdRP.

#### Investigation of possible cross-reactivity between CHIKV and MAYV seroprevalence

To exclude false-positive cases due to alphavirus cross-reactivity, all samples that were borderline or positive for anti-CHIKV IgG and had reminiscent specimens were tested for the presence of anti-MAYV IgG by ELISA. Among samples from the Vila Toninho participant study, we observed that one CHIKV-positive sample among tested ones (6.7%; 1/15) was positive for anti-MAYV IgG. Subsequently, we analyzed the anti-MAYV IgG-positive sample by PRNT_80_ and confirmed seroconversion to CHIKV and the absence of cross-reactivity with MAYV (S4 Table).

#### Investigation of anti-CHIKV neutralizing antibodies

**Fig 3:**
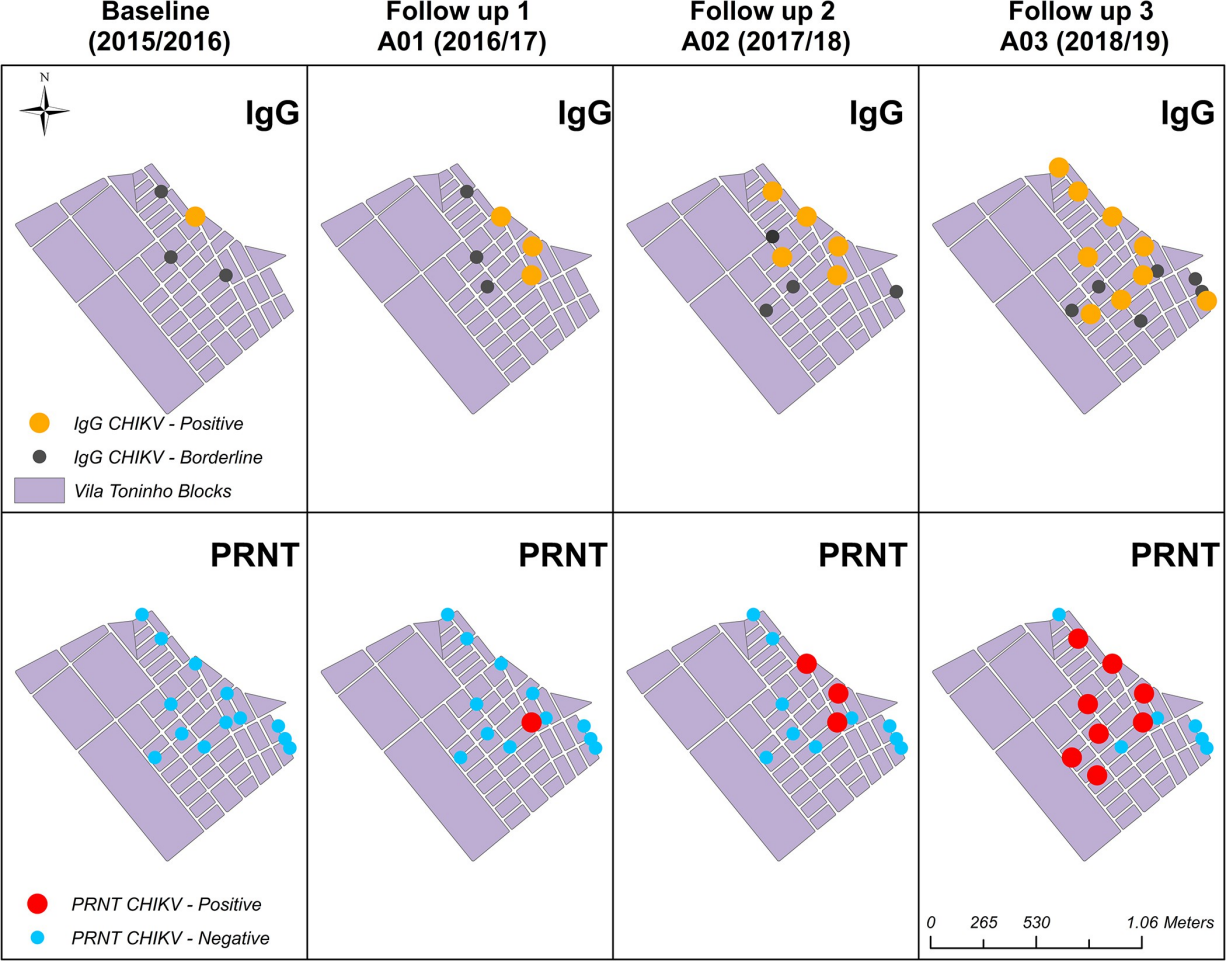
Spatial Distribution of Samples from Population Study According to Anti-CHIKV IgG and PRNT Assays. Thematic map showing the spatial distribution of positive (orange) and borderline positive (dark blue) serological results, according to anti-CHIKV IgG ELISA (A), and positive (red) or negative (blue) seroconversion samples from the population study, according to PRNT assays (B). Shapefile downloaded from https://portaldemapas.ibge.gov.br/portal.php#homepage.

Out of 15 paired samples positive or borderline by ELISA for anti-CHIKV IgG from the Vila Toninho study analyzed by PRNT_80_, 6.7% (n = 1/15) showed positivity for CHIKV-neutralizing antibodies in the first year. In contrast, in the last year, this rate increased to 46.7% (n = 7/15). The prevalence of positivity based on PRNT_80_ over the years was 0.3%, 0.58%, 0.87%, and 2.3%, slightly smaller than by IgG detection. Moreover, no individuals were positive for anti-MAYV IgG by PRNT_80_ assay (S4 Table). The comparison of anti-CHIKV IgG detection by ELISA and PRNT_80_ in Vila Toninho samples is presented in Fig 3. The individuals with the same results for anti-CHIKV antibodies detected by ELISA and PRNT_80_ confirmed that the participants were, in fact, exposed to CHIKV.

#### Vila Toninho’s entomological analysis

From October 2015 to August 2019, mosquito traps were placed in 2,830 houses to capture mosquitoes. A total of 7,960 mosquitoes were collected, of which 22.5% (1,755/7,960) were *A*. *aegypti*, 0.2% (14/7,960) were *A*. *albopictus*, and 77.3% (6,151/7,960) were *Culex sp*. Of the 1,795 *A*. *aegypti* identified, 34.9% (626/1,795) were males, which were divided into 350 pools, and 65.1% (1,169/1,795) were females, which were split into 701 pools (Table 3). Among the 13 pools of *A*. *albopictus*, 15.4% (2/13) were males, and 84.6% (11/13) were females.

**Table 3:** The Total Number of Aedes Species Pools Collected per Year of the Study (2015–2019).

Period	*Aedes aegypti ♂*			*Aedes aegypti ♀*			*Aedes albopictus ♂*			*Aedes albopictus ♀*		
	Total mosquitoes collected	Quantity of *pools*	% CHIKV-positive *(pools)*	Total mosquitoes collected	Quantity of *pools*	% CHIKV-positive *(pools)*	Total mosquitoes collected	Quantity of *pools*	% CHIKV-positive *(pools)*	Total mosquitoes collected	Quantity of pools	% CHIKV- positive *(pools)*
Baseline 2015/2016	86	54	4	246	168	15	0	0	0	5	5	2
Follow-up 1 2016/2017	153	88	0	263	159	0	0	0	0	4	3	0
Follow-up 2 2017/2018	195	113	0	381	214	0	0	0	0	3	3	0
Follow-up 3 2018/2019	192	95	**NA	279	160	**NA	2	2	**NA	0	0	**NA
Total	626	350	4	1.169	701	15	2	2	0	12	11	2

*The mosquito collection period was October and September each year.

**Not analyzed.

Out of 1,064 *Aedes* mosquito *pools*, 71.6% (762/1,064) were tested by RT–qPCR to identify circulating arboviruses. Among them, 2.76% (21/762) of *pools* were positive for CHIKV. Of the positive mosquito *pools*, 2.13% (15/701) were from female *A*. *aegypti*, and 1.43% (3/350) were from male *A*. *aegypti*, respectively. In addition, 18.18% (2/11) were female *pools* of *Ae*. *albopictus*. None were co-infected with other arboviruses was detected (Table 3). No *Culex* mosquitoes were tested for CHIKV.

**Fig 4:**
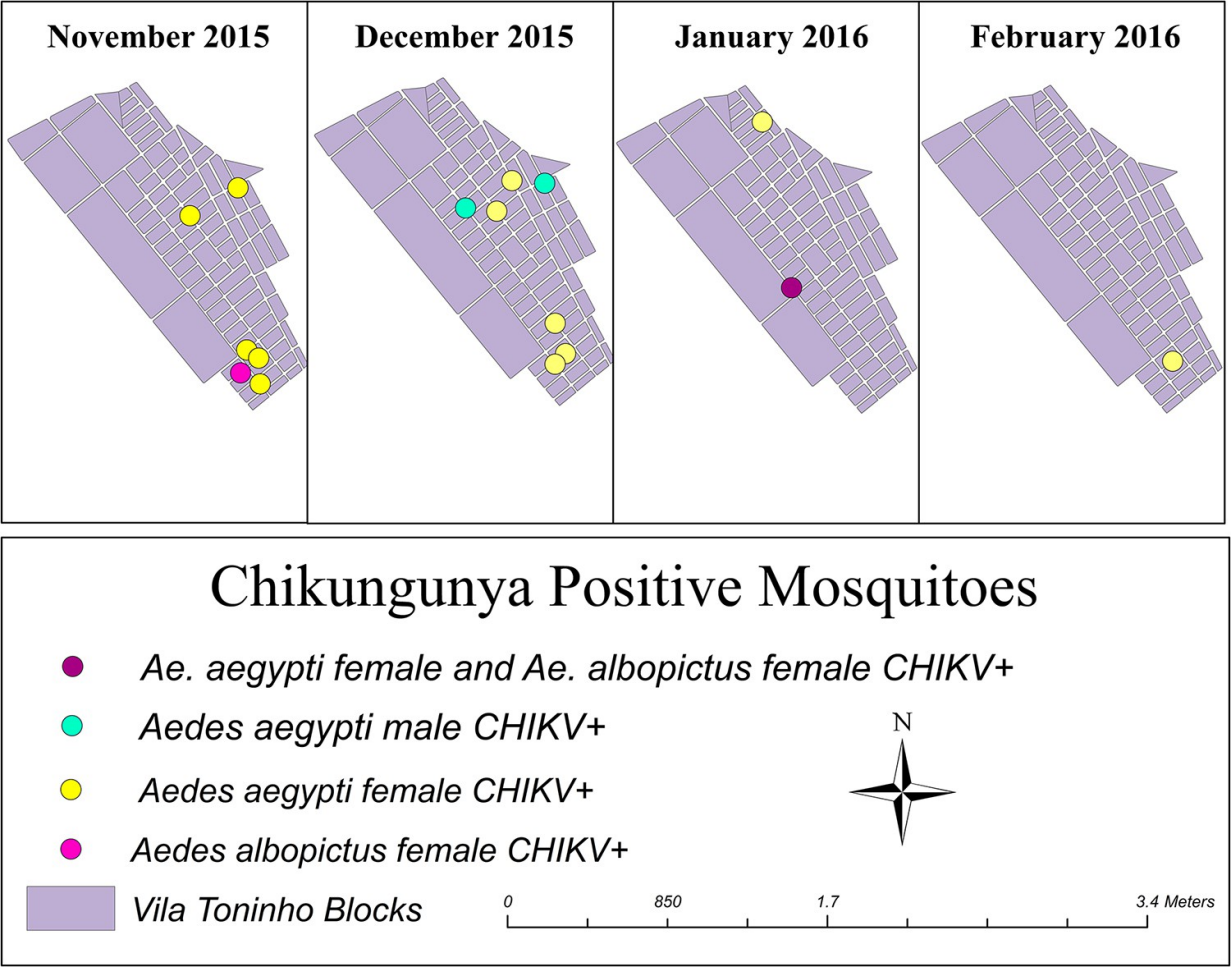
Spatial distribution of *A*. *albopictus* mosquitoes positive for CHIKV according to qRT–PCR in Vila Toninho, *SJdRP-SP*. Spatial distribution of *Ae*. *aegypti* and *Ae*. *albopictus* mosquitoes positive for CHIKV as detected by qRT–PCR (*Ae*. *aegypti* female and *Aedes albopictus* (purple), *Ae*. *aegypti* male (blue), *Ae*. *aegypti* female (yellow) and *Ae*. *albopictus* female (pink)) in this study, Vila Toninho, SJdRP-SP. Shapefile downloaded from https://portaldemapas.ibge.gov.br/portal.php#homepage.

To better understand the possible overlap of anti-CHIKV IgG-positive cases and *Aedes* mosquitoes positive for CHIKV, we built thematic maps showing the spatiotemporal relationship in the Vila Toninho neighborhood. Fig 4. shows the spatiotemporal distribution of mosquito pools positive for CHIKV. All positive pools were collected between November 2015 and February 2016, with January 2016 having the highest vector densities. After this period, every collected sample tested negative for CHIKV infection.

### The 2019 dengue outbreak and CHIKV circulation in SJdRP

In parallel to our prospective surveillance study in the Vila Toninho neighborhood, we extended our analyses to identify the circulation of CHIKV in other SJdRP regions using a panel of blood samples collected from dengue-suspected individuals during 2019, when a dengue epidemic was reported in the city. The presence of anti-CHIKV IgM and IgG antibodies was investigated in 497 samples out of 7,056 (7.0%), according to the eligibility criteria already described.

#### Detection of anti-CHIKV IgM and IgG in symptomatic patients

Among the 497 samples, 61.3% (305/497) were obtained from females, and 34.2% (170/497) were aged from 21 to 40 years. The prevalence of anti-CHIKV IgM was 4.4% (22/497, 95% CI 2.9–6.6), while 8.6% (43/497, 95% CI 6.5–11.4) of the samples were positive for anti-CHIKV IgG. Moreover, only one (0.2%) sample was positive simultaneously for anti-CHIKV IgM and IgG by ELISA. Interestingly, the 10-20-year-old age group presented the highest frequency of positivity for anti-CHIKV IgM (9.8%; 12/122; 95% CI 5.1–16.5, p<0.004) (Table 4).

**Table 4:** Anti-CHIKV IgG/IgM and anti-MAYV IgG in Panel Samples According to Sociodemographic Characteristics.

*Demographic variable*		*Epidemiological Surveillance 2019*										
		*Participants n (%)*	*Anti-CHIKV IgM (+)* *n (%)*	*95% CI*	*p value*	*Anti-CHIKV IgG (+)* *n (%)*	*95% CI*	*p value*	*Participants n (%)*	*Anti-MAYV IgG (+)* *n (%)*	*95% CI*	*p value*
	TOTAL	497 (100.0)	22 (4.4)	(2.9–6.6)		43 (8.6)	(0.3–2.6)		64 (100.0)	4 (6.2)	(1.7–15.2)	
	*SEX*											
	Male	192 (38.6)	13 (6.7)	(3.7–11.3)	0.1279	17 (8.9)	(5.2–13.8)	0.968	25 (39.0)	1 (4.0)	(1.6–20.9)	0.3882
	Female	305 (61.4)	9 (2.9)	(1.6–5.5)		26 (8.5)	(5.9–12.2)		39 (71.0)	3 (7.7)	(0.1–20.3	
*AGE (years)*												
	0–20	122 (24.7)	12 (9.8)	(5.1–16.5)	0.017	10 (8.2)	(4.0–14.6)	0.284	17 (XX)	0 (0.0)	0 (0.0)	0.178
	21–40	170 (34.2)	7 (4.1)	(1.7–8.3)		20 (11.8)	(7.3–17.6)		28 (43.7)	2 (7.1)	(0.9–23.5)	
	41–60	135 (27.1)	3 (2.2)	(0.5–6.4)		9 (6.7)	(3.1–12.3)		15 (23.4)	2 (13.3)	(1.7–40.5)	
	> 61	70 (14.0)	0 (0.0)	(0.0)		4 (5.7)	(1.6–14.0)		4 (6.3)	0 (0.0)	(0.0)	

Among the analyzed samples, 5.0% (25/497) belonged to residents from the Vila Toninho neighborhood, with 8.0% (2/25) positive for anti-CHIKV IgM and 4.0% (1/25) borderline positive for anti-CHIKV IgG. However, none of these individuals were participants in our prospective population study (study 1).

**Fig 5:**
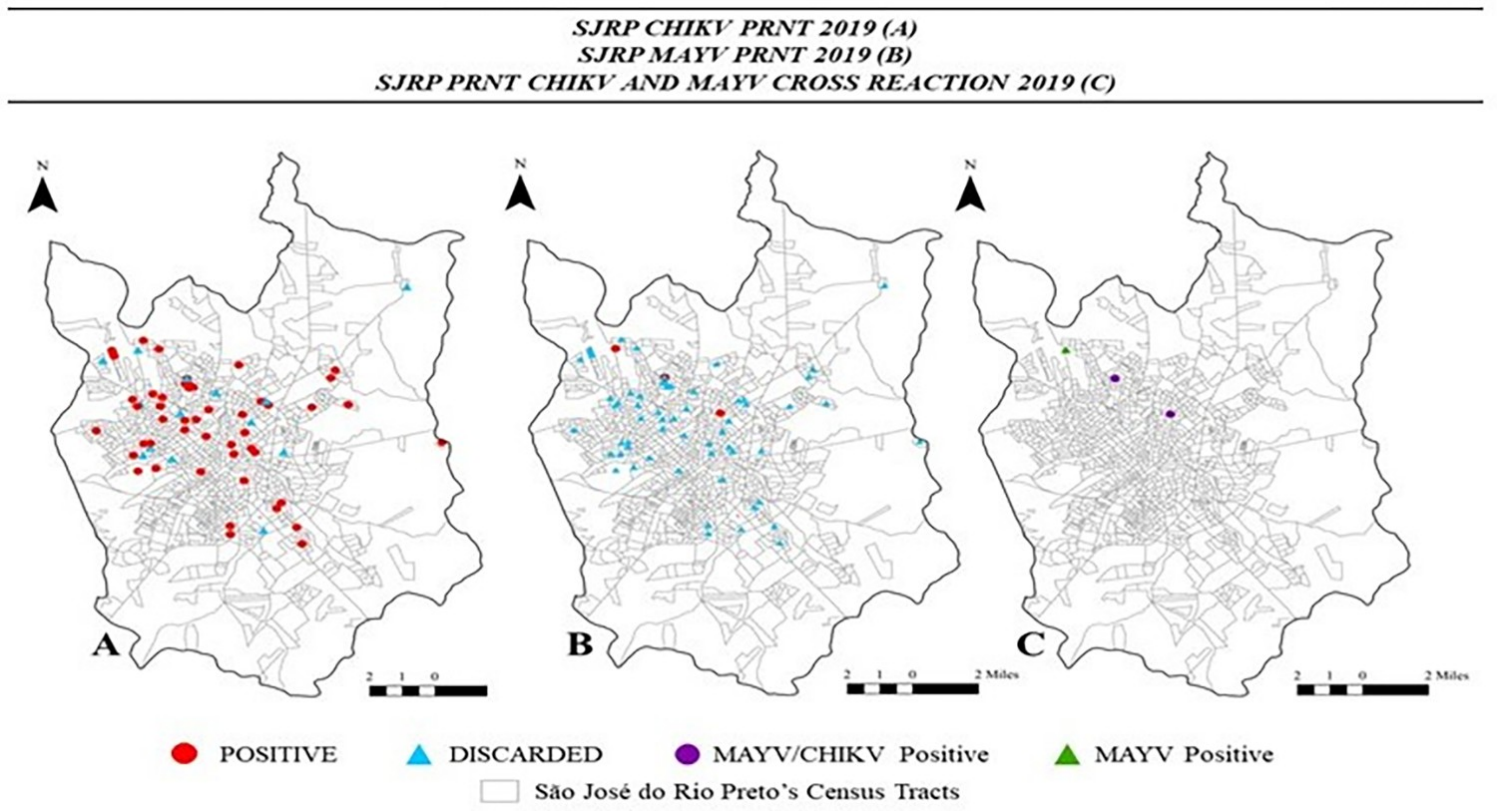
**Spatial Distribution of Samples According to Serological Status for CHIKV (A) and MAYV (B) by PRNT assay in 2019 during the dengue outbreak in São José do Rio Preto, SP.** Seroconversion results of patient samples in 2019 during the dengue outbreak in São José do Rio Preto, SP, illustrating the spatial distribution of serologic status by PRNT_90_ assay, which was positive (red) and negative (blue) for CHIKV (A) and MAYV (B) and positive (purple) for CHIKV/MAYV cross-reaction and positive (green) for MAYV (C). Shapefile downloaded from https://www.riopreto.sp.gov.br/mapas-rio-preto/.

Geospatial analysis showed that the distribution of individuals with anti-CHIKV IgM or IgG positivity occurred in all regions of SJdRP; however, it was more evident in the northern area, followed by the central district (Fig 5).

#### Association between symptoms and anti-CHIKV IgM positivity in samples from a panel of dengue-suspected patients

Among the panel of blood samples analyzed by ELISA, we collected clinical information from 65.4% (325/497) patients with one or more dengue-suspected symptoms. The data about signs and symptoms showed that the absence of retroorbital pain was significantly associated with anti-CHIKV IgM positivity (OR = 0.171, 95% CI 0.03–0.777, p<0.02). Other signs and symptoms did not present significant association with presence of anti-CHIKV IgM (S5 Table).

#### Investigation of anti-CHIKV neutralizing antibodies

The samples that were borderline or positive for anti-CHIKV IgG were tested for the presence of anti-MAYV IgG by ELISA. Among samples from the blood panel, 6.1% (4/65; 95% CI 0.3–20.0) were positive for anti-MAYV IgG. Subsequently, we analyzed those samples positive for anti-MAYV IgG by a PRNT_80_ assay to confirm seroconversion and the absence of cross-reactivity with MAYV (S6 Table).

Out of 65 blood samples from surveillance of dengue-suspected symptomatic patients tested by the PRNT_80_ assay for CHIKV and MAYV, 80.3% (n = 53) contained neutralizing antibodies to CHIKV, and three had neutralizing antibodies to MAYV. Among all the tested samples, only one was positive for MAYV, not CHIKV (Table 5). The complete information is available in S7 Table. The geospatial distribution of anti-CHIKV and anti-MAYV neutralizing antibodies was observed in regions with the highest population density in SJdRP (Fig 5).

**Table 5:** Anti-CHIKV IgG/IgM ELISA and Neutralizing Antibody Titers (PRNT_80_).

ID Blood Panel Sample	IgM_CHIKV ELISA	IgG_CHIKV ELISA	IgG_MAYV ELISA	CHIKV NEUT 80%	MAYV NEUT 80%	Anti-CHIKV titer PRNT_80_	Anti-MAYV titer PRNT_80_
2037	Negative	Positive	Positive	Negative	Positive	1:20	1:320
2128	Negative	Positive	Positive	Positive	Positive	1:40	1:160
2729	Positive	Negative	Negative	Positive	Negative	1:320	1:20

#### Detection and sequencing of CHIKV samples from patients with acute disease

Finally, the samples from symptomatic CHIKV-confirmed patients were analyzed to investigate the circulating CHIKV genotype from 2015 to 2020. Among 41 samples tested for the envelope genome region (E1/6K/E2) of CHIKV by qRT–PCR, three were submitted to sequencing analysis due to their Ct values, and only one yielded an envelope sequence.

**Fig 6:**
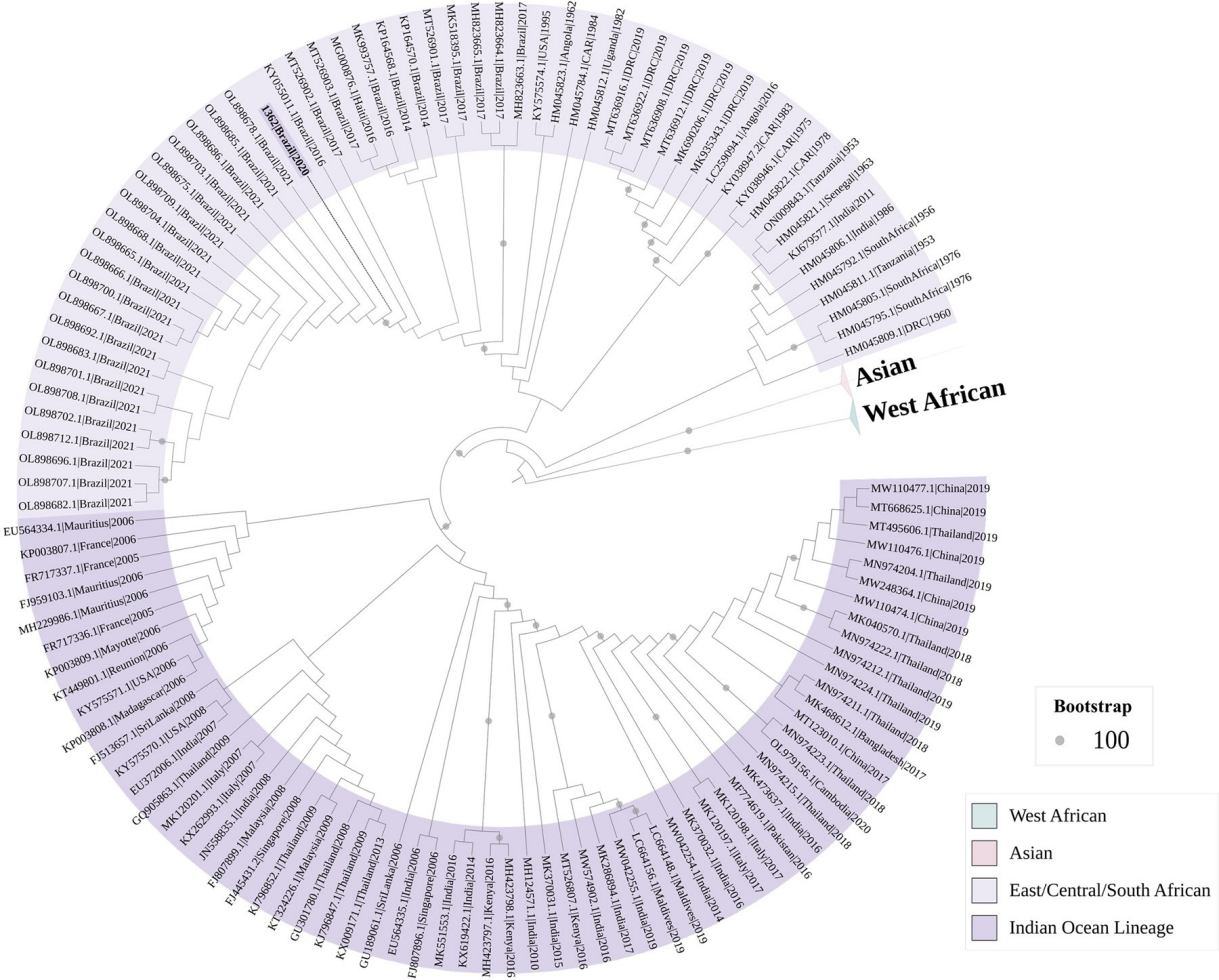
Maximum likelihood tree of chikungunya virus based on partial envelope gene sequence. Phylogenetic tree reconstructed using the Maximum-likelihood method with TIM2e+4 as nucleotide substitution model, using Ultrafast Bootstrap (UFBoot) combined with SH-like Approximate Likelihood-ratio test (SH-aLRT). The analysis involved 172 nucleotide (nt) sequences (1,885 nt). Branch lengths are drawn to a scale of nucleotide substitutions per site according to the scale. The analysis was conducted in IQ-TREE v. 2.0.3, and the final tree was visualized and edited in iTOL v. 6.6. The sequence from this study (1362|Brazil|2020) is highlighted in purple within the East/Central/South African genotype clade.

The sequenced sample was collected from a 60-year-old female patient who reported fever, myalgia, headache, back pain, nausea, and severe arthralgia, with no travel history in the 30 days before symptom onset. She was a resident of the impoverished area near the Piedade stream, SJdRP. The sequences were grouped within the ECSA lineage; however, we did not identify any of the E1/E2 albopictus-adaptative mutations which CHIKV transmission, as described by Tsetsarkin et al. (2014) (Fig 6) [42].

## Discussion

Here, we performed the first epidemiological study of CHIKV circulation in SJdRP through serological analysis of the population, molecular detection of the virus in mosquitoes, and sequencing and phylogenetic analyses, showing the circulation of the Central-East-South Africa (ECSA) lineage in the city. Unfortunately, as it was not possible to sequence more samples due to the low viral load in mosquitoes and the scarcity of the original sample, we cannot state that the ECSA lineage is the predominant one in the region. The spread of CHIKV has occurred in epidemic waves with large magnitudes [43,44]. Unlike the natural history of the disease in most of Brazil [6], cryptic dissemination of CHIKV in a population widely exposed to other arboviruses occurred in SJdRP, without a notable outbreak but by inapparent cases that could be detected only through systematic active surveillance. We observed an increase in the prevalence of anti-CHIKV IgG antibodies over four years in individuals who were apparently asymptomatic. In addition, we detected anti-CHIKV IgM antibodies, a common marker of recent infection, in symptomatic patients who were discharged during a 2019 dengue outbreak.

In our study, the overall CHIKV seroprevalence observed was 2.1%, and the global incidence was 20.6 cases/1,000 inhabitants based on IgG ELISA seroreactivity, which is considered extremely low when compared to that in studies conducted in areas that were epicenters of large CHIKV epidemics in Brazil [6,44,45] or in several other countries [43,46–51]. The investigation based on PRNT_80_, the gold-standard method, showed data very close. However, our data are divergent from those reported by municipal bulletins, in which the overall incidence of CHIKV infections (0.44/1,000 inhabitants) was still lower from 2015 to 2020 [39]. All these findings corroborate that CHIKV infection has been underdiagnosed in the city, and its circulation has been cryptic over the last five years.

A study conducted in other Brazilian cities showed different of seroprevalence rates: 42.3% in Feira de Santana and 30.9% in Riachão do Jacuípe [44]. In a serosurvey study in India, the overall CHIKV seroprevalence was 18.1%, with heterogeneity among age groups and geographic regions [52]. However, both studies analyzed anti-CHIKV IgG seroprevalence in populations that had been facing an outbreak. This silent CHIKV circulation has already been described in areas that have experienced epidemics after this scenario, in which asymptomatic cases were detected through the presence of anti-CHIKV IgG in a substantially higher number than symptomatic acute cases [18,53–56]. These data can represent a warning to the health authorities of SJdRP and other places in Brazil and in the world where CHIKV has been introduced. As shown by the analysis of the prospective study, approximately 98% of participants were still vulnerable to infection by CHIKV after five years of cryptic viral circulation, which could represent a risk of an epidemic caused by the virus.

The investigation by PRNT_80_ assay has shown that not all individuals with anti-CHIKV IgG antibodies detected by ELISA had neutralizing antibodies. Among patients who presented anti-CHIKV IgG positivity or borderline positivity during follow-up, only 6.7% were positive for CHIKV-neutralizing antibodies. This rate increased to 46.7% in the last year of follow-up and was even higher in panel blood samples, reaching 80.3%. A study in India showed different neutralizing antibody rates, 58.3% Delhi and 13.5% in Mumbai [57], while in Kenya, the CHIKV-neutralizing antibody rate was 0.7 to 5.2% [58]. In Salvador, in a randomized study two years after a CHIKV epidemic, the neutralizing antibody rate was 90.0% [17]. The high presence of neutralizing antibodies was demonstrated in a CHIKV cases cohort in the Philippines, which showed levels of around 100.0%. [18,59].

Due to the possibility of cross-reaction between alphaviruses, we performed experiments to detect anti-MAYV IgG antibodies. The presence of MAYV-neutralizing antibodies was not detected in any Vila Toninho study sample, while two samples from the blood panel contained CHIKV- and MAYV-neutralizing antibodies. MAYV is endemic in the Amazon Basin in the northern Brazilian region, although it has been detected in additional regions [60–62] due to the mobility of people. The virus is not known to circulate in SJdRP, but an imported MAYV case has already been described in the municipality [63,64]. Thus, individuals detected as MAYV-positive in our study might have been exposed to this virus previously or in another locality since there is no autochthonous transmission of MAYV in SJdRP. However, due to a lack of paired samples and information about recent travel history, no further conclusions could be reached. On the other hand, this finding indicates the importance of active surveillance to monitor the introduction of new viruses.

Geospatial analysis showed a distribution of individuals positive for anti-CHIKV IgM or IgG across all regions of SJdRP; however, it was more evident in the northern area, followed by the central district. Coincidentally, these areas have the highest population density in the municipality of SJdRP. Moreover, the fact that patients carry anti-CHIKV IgM antibodies in all areas of the city suggests active virus circulation and the risk of a future epidemic.

Among the correlated sociodemographic factors, only age was significantly associated with the presence of anti-CHIKV IgM antibodies, as also demonstrated in epidemiological studies in other countries [17,43,44,65]. As individuals age and transition into adulthood, their increased mobility for educational and professional purposes exposes them to multiple locations, including areas infested by *Aedes* mosquitoes. In addition, more-active people tend to have a greater aura of CO_2_, which would attract mosquitoes [43,66]. Although women were more prevalent in this study, sex was not associated with the presence of CHIKV antibodies, which was observed during studies conducted in El Salvador [17,67]. However, other studies have reported the presence of IgM/IgG antibodies is associated to female sex, as well as long-term manifestation of CHIKV are more prevalent in individuals with preexisting rheumatic diseases, such as any form of rheumatoid arthritis [44,65,68]. In contrast, the male sex was related to exposure to the vector for long periods [69].

Overlapping symptoms triggered by different arboviruses can be a complicating factor when defining the differential diagnosis, especially in areas of virus cocirculation. This fact was apparent when we evaluated the signs and symptoms reported by dengue-suspected patients who had this diagnosis excluded and presented anti-CHIKV IgM positivity. Retroorbital pain was the most frequently associated symptom with a lower chance of anti-CHIKV IgM antibody positivity. The symptom is well known to be characteristic of dengue. This result reflects the importance of performing specific diagnostic tests for etiological definition. Furthermore, other signs and symptoms did not show an association with either of the diseases, demonstrating that they may not be indicative of one infection or another and, therefore, cannot be used as clinical factors for differentiation. They are tools that allow not only the structuring of the health network to manage specific diseases but also generate safety for the targeted population and adequate clinical management by health professionals in areas of agent cocirculation [70]. Due to the high similarity of symptoms among arboviruses, specific diagnostic tests should be implemented along with a careful analysis of the patient in order to discriminate the actual cases, thus decreasing the number of underreported CHIKV or classified as another arbovirus.

Although CHIKV was sequenced in only one sample, we were able to identify the ECSA lineage of CHIKV as circulating in SJdRP, grouped with samples identified since 2016 in the country [71,72]. Notably, our study was based on serological findings for CHIKV, and the antibody-based immune response to an agent is usually triggered 5–7 days after the onset of symptoms, a period that coincides with natural viral clearance [73], which could limit CHIKV RNA detection. The ECSA lineage has been established as responsible for CHIKV infection cases in different regions of Brazil [6,45,74–80], mainly in the Northeast Region, which was the epicenter of the CHIKV epidemic in the country [6,81] and the Southeast Region, with outbreaks in the states of Rio de Janeiro, São Paulo and Minas Gerais [72,77,82–86].

Phylogenetic characterization studies contributed to understanding how and why the magnitude of CHIKV infection cases has been so disproportionate in the transmission dynamics of the disease in the population. The emergence of new CHIKV strains responsible for recent epidemics demonstrates that CHIKV genotypes carry diverse mutations. Such mutations can provide advantages and disadvantages that alter the infectivity, transmission, and adaptability dynamics of CHIKV, resulting in a higher vectorial capacity for transmission in naïve populations or in areas already endemic for CHIKV [1]. On the other hand, undetected mutations may exist in the viral population, as reported during a phylogenetic characterization study of CHIKV during an epidemic in SP [72]. The limitation of not fully sequencing the virus in the vector and the samples impeded from determining whether unknown mutations would have altered the modulation in the interaction of CHIKV with different vectors and influenced the dynamics of virus dispersion among the population, where CHIKV was introduced and not causing epidemics, but maintaining continuous circulation, as evidenced in our study by the presence of asymptomatic individuals with anti-CHIKV antibodies. These mutations may be a direction for studies to answer the questions about how these genetic mutations impact infection dynamics.

The entomological analysis confirmed the presence of CHIKV in both *A*. *aegypti* and *A*. *albopictus* mosquitoes in domestic and peridomestic areas. Although a pool of *A*. *albopictus* females was positive for CHIKV, we cannot infer its role in the dispersion of the virus, as *Ae albopictus* has no domestic habituation, and the presence of CHIKV might be related only to previous meals. This finding allows us to infer only the flow of the virus in the vector and CHIKV circulation among humans in the same area. Notably, until now, the natural presence of CHIKV was associated only with *A*. *aegypti* in Brazil, and our detection of CHIKV in *A*. *albopictus* is the first report in the country. Here, we were not able to characterize the CHIKV genotype present in both mosquitoes due to low viral load in the sample from the mosquito pools, as reported in studies conducted in Gabon and Thailand [87,88]. Even so, the presence of the CHIKV ECSA genotype has been naturally associated with *A*. *aegypti* mosquitoes, as characterized by Costa-da-Silva et al. [71].

The likelihood of a virus emerging in a naive population depends on its ability to adapt to new hosts. Understanding and predicting the occurrence of adaptative mutations in arboviruses may be critical to mitigate potential future outbreaks [89]. Between 2007–2009 an important example was documented in India showing how one mutation can affect CHIKV transmission [90]. The acquisition of the A226V mutation in the CHIKV-E1 protein might influence the viral fitness, infectivity, and transmission in *A*. *albopictus* [91].

In Brazil, CHIKV cases have been linked to *A*. *aegypti* as a vector. Besides, we did not identify any E1/E2 albopictus-adaptative mutations, but the concern about potential CHIKV outbreaks remains. Our detection of CHIKV in male *A*. *aegypti* mosquitoes suggests active viral circulation once the viral presence in male mosquitoes is associated with vertical transmission or by infected female mosquitoes during copulation. It may explain in part the cryptic CHIKV circulation in the SJdRP population. In part, because the vectorial competence of different CHIKV lineages may vary according to mosquitoes species and population features, which become the spread of lineage particular in each region and susceptible to other viruses transmission, such as DENV and ZIKV [35,92,93].

CHIKV and ZIKV were notably introduced at almost the same time in SJdRP, reflecting the scenario of several cities in Brazil that also experienced DENV outbreaks in 2015 [94]. Importantly, between 2015 and 2018, the municipality suffered from a ZIKV epidemic [95–97], and in 2019, it experienced the largest DENV epidemic ever recorded [39], in contrast to the lack of a CHIKV epidemic. This dichotomy in experiences for CHIKV outbreaks, which were different for the other regions of the country, derives from a complex multifactorial phenomenon that involves the interaction between virus and host and vector competence, which modulate the disease and the dynamics of simultaneous transmission of different arboviruses. External factors such as meteorological conditions, food availability, urban mobility, and ecosystem change act directly on vector density and behavior, and the consonance of a population without preexisting immunity favors the emergence and re-emergence of new arboviruses [98,99].

Our study had limitations, especially in the logistical aspects of maintaining a long-term prospective population study. The high number of participants who changed addresses or phone numbers or were absent during follow-up home visits reduced the number of participants with paired samples throughout the study. Even so, more than 300 participants were entirely followed for four years. Besides, the sequence obtained from CHIKV was not the whole genome, which hindered our understanding of potential mutations and changes in virulence. Investigating these gaps in the dynamics of CHIKV dissemination and other arboviruses, as well as potential mutations favoring replication in both humans and mosquitoes, is essential. Identification of the circulating CHIKV genotype among the vector population was not possible for two reasons: *i*) a low viral load was detected in the *A*. *aegypti* and *A*. *albopictus* mosquitoes collected at baseline in the entomological study in 2015 [92], and *ii*) the pools collected in 2019 were preferably used for studies of DENV and ZIKV since the municipality experienced the largest DENV epidemic [39]. Finally, the low viral load or absence of CHIKV in the samples from symptomatic patients did not allow us to determine whether the ECSA genotype was responsible for the majority of CHIKV infections in the city since its introduction and whether it maintained ECSA circulation or if this genotype was reintroduced, as demonstrated in studies in Bahia and Rio Grande do Norte [53,79]. It was possible to identify it as a genotype present in 2019, but no conclusions about its circulation dynamics could be made. This was a limitation initially assumed in the study when the inclusion and exclusion criteria were established, and we opted for the model based on serological research results as diagnostic criteria for CHIKF.

In conclusion, our study identified that although CHIKV was not responsible for epidemics in SJdRP, it is present and, unlike dengue, circulates only at low levels without apparent epidemics. We, therefore, confirmed its cryptic circulation in the city, highlighting the presence of asymptomatic individuals with anti-CHIKV antibodies, despite active participant monitoring. Thus, this study suggests that although the virus has not yet demonstrated its impact through epidemics or even during interepidemic periods, cryptic CHIKV circulation in the population should be strongly considered. The cryptic circulation of CHIKV may precede epidemics and may have an immeasurable impact on public health, mainly due to the high prevalence of symptomatic cases associated with comorbidities and morbidity. Implementing of epidemiological sentinel studies with clinical, entomologic, and genomic interrelation has become increasingly indispensable worldwide. The underdiagnosed asymptomatic cases, when not actively investigated as in this study, demonstrate how the disease may be neglected. These factors impact viral dynamics and make it difficult to understand how the natural history of CHIKF has evolved so diversely throughout the countries where it has been identified, especially in areas where other arboviruses are cocirculating and are present in *A*. *aegypti* and *A*. *albopictus* mosquitoes as vectors responsible for the transmission of these diseases. Such a circulation profile does not minimize its potential as a public health threat due to the individual impact of morbidity, which will require structuring a health network for such patients, and the risk of future epidemics.

## Supporting information

S1 Table Primers used in the sequencing of the structural genes (E1/6k/E2) of CHIKV. (DOCX)

S2 Table Information of chikungunya virus (CHIKV) sequences included in the dataset. (DOCX)

S3 Table Sociodemographic variables of the population study between 2015 and 2019. (DOCX)

S4 Table Results of seropositivity to CHIKV antibodies, according with anti-CHIKV IgG by ELISA assay and neutralizing antibody titers (PRNT_80_) for the participant paired samples from Vila Toninho during the study (FB = Baseline 2015/2016, A01 = Follow-up 2016/2017, A02 = Follow-up 2017/2018 and A03 = Follow-up 2018/2019). (DOCX)

S5 Table Association of the presence or absence of symptoms and positive and negative status for CHIKV IgM antibodies determined by ELISA among dengue-suspected patients during the 2019 dengue outbreak. (DOCX)

S6 Table Serological outcome of the anti-CHIKV IgG/IgM (ELISA) and neutralizing antibody titers (PRNT_80_) in samples from dengue-suspected patients in the 2019 outbreak. (DOCX)

S7 Table The over dilution samples from dengue-suspected patients in the 2019 outbreak, which supported CHIKV and MAYV cross-reaction during neutralizing antibody titration (PRNT_80_). (DOCX)

S1 Dataset The database used in this manuscript. (XLSX)
