# Supplementary material for: Cryptic circulation of chikungunya virus in São Jose do Rio Preto, Brazil, 2015–2019
Source: PLoS Negl Trop Dis. 2024 Mar 14;18(3):e0012013. doi: 10.1371/journal.pntd.0012013 (PMC10965090; doi:10.1371/journal.pntd.0012013)
Supplement: S1 Table — (DOCX) [file pntd.0012013.s001.docx]

**S1 Table. Primers used for sequencing of the envelope structural genes (E1/6k/E2) of CHIKV.**

|  | **Primer** | **Sequence (5’ >> 3’)** | **Position*** | **Fragment (bp)** |
| --- | --- | --- | --- | --- |
|  | 1F | TGAGCCCCGGGTACTATCAG | 8482-8501 | 604 |
|  | **1R** | **AATGTGCGATCTGGGGTGTC** | **9066-9085** |  |
|  | 2F | AGGGAACTACCTTGCAGCAC | 8991-9010 | 588 |
|  | **2R** | **TGTGGATAACTGCGGCCAAT** | **9559-9578** |  |
|  | 3F | AGATCAGGTTAACCGTGCCG | 9493-9512 | 596 |
|  | **3R** | **CTCCAATACCATGGGGCTGT** | **10069-10088** |  |
|  | 4F | ATGCAAGCCCTTATTCCGCT | 9874-9892 | 569 |
|  | **4R** | **TGCATAAGCAGCCACAGTGA** | **10423-10442** |  |
|  | 5F | CAGGGCTCATACCGCATCCG | 10364-10383 | 591 |
|  | **5R** | **TTAATGATGGCTACGCCCCC** | **10935-10954** |  |
|  | 6F | GGTATCAGCCTGCACCCATT | 10904-10923 | 634 |
|  | **6R** | **GGGGTTGTTCAGCCCTTAGA** | **11518-11537** |  |

*Position in the genome established according to SINAN (Information System for Notifiable Diseases) data and comparison with the sequence of CHIKV (*Chikungunya virus* isolate C302F/2016/BR, complete genome*,* GenBank access number KY055011.1).
