# Supplementary material for: Cryptic circulation of chikungunya virus in São Jose do Rio Preto, Brazil, 2015–2019
Source: PLoS Negl Trop Dis. 2024 Mar 14;18(3):e0012013. doi: 10.1371/journal.pntd.0012013 (PMC10965090; doi:10.1371/journal.pntd.0012013)
Supplement: S2 Table — (DOCX) [file pntd.0012013.s002.docx]

**S2 Table. Information of chikungunya virus (CHIKV) sequences included in the dataset.**

| **GenBank ID** | **Country** | **Continent** | **Year** |
| --- | --- | --- | --- |
| 1362 | Brazil | South America | 2020 |
| AY726732.1 | Senegal | Africa | 1983 |
| EU372006.1 | India | Asia | 2007 |
| EU564334.1 | Mauritius | Africa | 2006 |
| EU564335.1 | India | Asia | 2006 |
| FJ445431.2 | Singapore | Asia | 2008 |
| FJ513657.1 | Sri Lanka | Asia | 2008 |
| FJ807896.1 | Singapore | Asia | 2006 |
| FJ807899.1 | Malaysia | Asia | 2008 |
| FJ959103.1 | Mauritius | Africa | 2006 |
| FR717336.1 | France | Europe | 2005 |
| FR717337.1 | France | Europe | 2005 |
| GQ905863.1 | Thailand | Asia | 2009 |
| GU189061.1 | Sri Lanka | Asia | 2006 |
| GU301780.1 | Thailand | Asia | 2008 |
| HE806461.1 | New Caledonia | Oceania | 2011 |
| HM045784.1 | Central African Republic | Africa | 1984 |
| HM045785.1 | Senegal | Africa | 1966 |
| HM045786.1 | Nigeria | Africa | 1964 |
| HM045787.1 | Thailand | Asia | 1995 |
| HM045788.1 | India | Asia | 1973 |
| HM045791.1 | Indonesia | Asia | 1983 |
| HM045792.1 | South Africa | Africa | 1956 |
| HM045795.1 | South Africa | Africa | 1976 |
| HM045798.1 | Senegal | Africa | 1966 |
| HM045800.1 | Philippines | Asia | 1985 |
| HM045803.1 | India | Asia | 1963 |
| HM045805.1 | South Africa | Africa | 1976 |
| HM045806.1 | India | Asia | 1986 |
| HM045809.1 | Democratic Republic of the Congo | Africa | 1960 |
| HM045810.1 | Thailand | Asia | 1958 |
| HM045811.1 | Tanzania | Africa | 1953 |
| HM045812.1 | Uganda | Africa | 1982 |
| HM045814.1 | Thailand | Asia | 1975 |
| HM045815.1 | Senegal | Africa | 1979 |
| HM045816.1 | Senegal | Africa | 1966 |
| HM045817.1 | Senegal | Africa | 2005 |
| HM045818.1 | Cote d'Ivoire | Africa | 1981 |
| HM045819.1 | Senegal | Africa | 1993 |
| HM045820.1 | Cote d'Ivoire | Africa | 1993 |
| HM045821.1 | Senegal | Africa | 1963 |
| HM045822.1 | Central African Republic | Africa | 1978 |
| HM045823.1 | Angola | Africa | 1962 |
| JN558835.1 | India | Asia | 2008 |
| KJ679577.1 | India | Asia | 2011 |
| KJ796847.1 | Thailand | Asia | 2009 |
| KJ796852.1 | Thailand | Asia | 2009 |
| KM923920.1 | Malaysia | Asia | 2007 |
| KP003807.1 | France | Europe | 2006 |
| KP003808.1 | Madagascar | Africa | 2006 |
| KP003809.1 | Mayotte | Africa | 2006 |
| KP164568.1 | Brazil | South America | 2014 |
| KP164570.1 | Brazil | South America | 2014 |
| KR046228.1 | Trinidad and Tobago | South America | 2014 |
| KR046233.1 | Trinidad and Tobago | South America | 2014 |
| KR559471.1 | El Salvador | Central America | 2014 |
| KT324226.1 | Malaysia | Asia | 2009 |
| KT449801.1 | Reunion | Africa | 2006 |
| KX009171.1 | Thailand | Asia | 2013 |
| KX168429.1 | Malaysia | Asia | 2009 |
| KX262986.1 | Senegal | Africa | 1983 |
| KX262987.1 | Thailand | Asia | 1996 |
| KX262988.1 | Thailand | Asia | 1988 |
| KX262993.1 | Italy | Europe | 2007 |
| KX262995.1 | Senegal | Africa | 1983 |
| KX496989.1 | Colombia | South America | 2016 |
| KX619422.1 | India | Asia | 2014 |
| KX702402.1 | Haiti | North America | 2014 |
| KY038946.1 | Central African Republic | Africa | 1975 |
| KY038947.2 | Central African Republic | Africa | 1983 |
| KY055011.1 | Brazil | South America | 2016 |
| KY575570.1 | USA | North America | 2008 |
| KY575571.1 | USA | North America | 2006 |
| KY575574.1 | USA | North America | 1995 |
| KY680348.1 | USA | North America | 2014 |
| KY680361.1 | USA | North America | 2014 |
| KY680366.1 | USA | North America | 2015 |
| KY680379.1 | USA | North America | 2014 |
| KY680384.1 | USA | North America | 2015 |
| KY680391.1 | USA | North America | 2014 |
| KY680407.1 | USA | North America | 2014 |
| KY703913.1 | Nicaragua | Central America | 2015 |
| KY703914.1 | Nicaragua | Central America | 2015 |
| KY703918.1 | Nicaragua | Central America | 2015 |
| KY703922.1 | Nicaragua | Central America | 2015 |
| KY703923.1 | Nicaragua | Central America | 2015 |
| KY703949.1 | Nicaragua | Central America | 2015 |
| KY703957.1 | Nicaragua | Central America | 2015 |
| KY703968.1 | Nicaragua | Central America | 2015 |
| KY703974.1 | Nicaragua | Central America | 2015 |
| KY703998.1 | Nicaragua | Central America | 2015 |
| LC259083.1 | Indonesia | Asia | 2009 |
| LC259084.1 | Philippines | Asia | 2012 |
| LC259085.1 | Indonesia | Asia | 2012 |
| LC259094.1 | Angola | Africa | 2016 |
| LC664148.1 | Maldives | Asia | 2019 |
| LC664156.1 | Maldives | Asia | 2019 |
| MF001510.1 | USA | North America | 2015 |
| MF773565.1 | Timor-Leste | Asia | 2010 |
| MF774619.1 | Pakistan | Asia | 2016 |
| MG000876.1 | Haiti | North America | 2016 |
| MG664851.1 | China | Asia | 2012 |
| MH124571.1 | India | Asia | 2010 |
| MH229986.1 | Mauritius | Africa | 2006 |
| MH423797.1 | Kenya | Africa | 2016 |
| MH423798.1 | Kenya | Africa | 2016 |
| MH670649.1 | Malaysia | Asia | 2009 |
| MH823663.1 | Brazil | South America | 2017 |
| MH823664.1 | Brazil | South America | 2017 |
| MH823665.1 | Brazil | South America | 2017 |
| MK040570.1 | Thailand | Asia | 2018 |
| MK120197.1 | Italy | Europe | 2017 |
| MK120198.1 | Italy | Europe | 2017 |
| MK120201.1 | Italy | Europe | 2007 |
| MK286894.1 | India | Asia | 2017 |
| MK286896.1 | India | Asia | 2017 |
| MK370031.1 | India | Asia | 2015 |
| MK370032.1 | India | Asia | 2016 |
| MK468612.1 | Bangladesh | Asia | 2017 |
| MK473637.1 | India | Asia | 2016 |
| MK518395.1 | Brazil | South America | 2017 |
| MK551553.1 | India | Asia | 2016 |
| MK690206.1 | Democratic Republic of the Congo | Africa | 2019 |
| MK935343.1 | Republic of the Congo | Africa | 2019 |
| MK993757.1 | Brazil | South America | 2016 |
| MN974204.1 | Thailand | Asia | 2019 |
| MN974211.1 | Thailand | Asia | 2018 |
| MN974212.1 | Thailand | Asia | 2019 |
| MN974215.1 | Thailand | Asia | 2018 |
| MN974222.1 | Thailand | Asia | 2018 |
| MN974223.1 | Thailand | Asia | 2018 |
| MN974224.1 | Thailand | Asia | 2019 |
| MT123010.1 | China | Asia | 2017 |
| MT495606.1 | Thailand | Asia | 2019 |
| MT526807.1 | Kenya | Africa | 2016 |
| MT526901.1 | Brazil | South America | 2017 |
| MT526902.1 | Brazil | South America | 2017 |
| MT526903.1 | Brazil | South America | 2017 |
| MT636908.1 | Democratic Republic of the Congo | Africa | 2019 |
| MT636912.1 | Democratic Republic of the Congo | Africa | 2019 |
| MT636916.1 | Democratic Republic of the Congo | Africa | 2019 |
| MT636922.1 | Democratic Republic of the Congo | Africa | 2019 |
| MT668625.1 | China | Asia | 2019 |
| MW042254.1 | India | Asia | 2014 |
| MW042255.1 | India | Asia | 2019 |
| MW110474.1 | China | Asia | 2019 |
| MW110476.1 | China | Asia | 2019 |
| MW110477.1 | China | Asia | 2019 |
| MW248364.1 | China | Asia | 2019 |
| MW574902.1 | India | Asia | 2016 |
| OK316992.1 | China | Asia | 2019 |
| OK316996.1 | China | Asia | 2020 |
| OL898665.1 | Brazil | South America | 2021 |
| OL898666.1 | Brazil | South America | 2021 |
| OL898667.1 | Brazil | South America | 2021 |
| OL898668.1 | Brazil | South America | 2021 |
| OL898675.1 | Brazil | South America | 2021 |
| OL898678.1 | Brazil | South America | 2021 |
| OL898682.1 | Brazil | South America | 2021 |
| OL898683.1 | Brazil | South America | 2021 |
| OL898685.1 | Brazil | South America | 2021 |
| OL898686.1 | Brazil | South America | 2021 |
| OL898692.1 | Brazil | South America | 2021 |
| OL898696.1 | Brazil | South America | 2021 |
| OL898700.1 | Brazil | South America | 2021 |
| OL898701.1 | Brazil | South America | 2021 |
| OL898702.1 | Brazil | South America | 2021 |
| OL898703.1 | Brazil | South America | 2021 |
| OL898704.1 | Brazil | South America | 2021 |
| OL898707.1 | Brazil | South America | 2021 |
| OL898708.1 | Brazil | South America | 2021 |
| OL898709.1 | Brazil | South America | 2021 |
| OL898712.1 | Brazil | South America | 2021 |
| OL979156.1 | Cambodia | Asia | 2020 |
| ON009843.1 | Tanzania | Africa | 1953 |

ID: identification. CHIKV: chikungunya virus. This dataset included 173 CHIKV nucleotide (nt) sequences, spanning 1,885 nt.
