# Supplementary material for: Cryptic circulation of chikungunya virus in São Jose do Rio Preto, Brazil, 2015–2019
Source: PLoS Negl Trop Dis. 2024 Mar 14;18(3):e0012013. doi: 10.1371/journal.pntd.0012013 (PMC10965090; doi:10.1371/journal.pntd.0012013)
Supplement: S3 Table — (DOCX) [file pntd.0012013.s003.docx]

**S3 Table. Sociodemographic variables of the population subcohort study between 2015 and 2019.**

|  |  |  |
| --- | --- | --- |
| Sociodemographic characteristics |  | Participant n (%) |
|  | TOTAL | 341 (100.0) |
| SEX |  |  |
|  | Male | 126 (37.0) |
|  | Female | 215 (63.0) |
|  |  |  |
| ETHNICITY |  |  |
|  | White | 198 (58.0) |
|  | Black | 38 (11.1) |
|  | Brown and others | 101 (29.6) |
|  | Missing | 4 (1.1) |
| AGE (years) | |  |
|  | 10-20 | 17 (5.0) |
|  | 21-40 | 65 (19.0) |
|  | 41-60 | 141 (41.3) |
|  | > 61 | 118 (34.6) |
| SCHOOLING (years of study) | | |
|  | 0-2 | 44 (12.9) |
|  | 3-7 | 161 (47.2) |
|  | 8-11 | 109 (31.9) |
|  | > 12 | 25 (7.3) |
| PER CAPITA INCOME (R$) | |  |
|  | 0.00-980.00 | 43 (12.6) |
|  | 981.00–3,000.00 | 181 (53.0) |
|  | 3,001.00–7,000.00 | 70 (17.0) |
|  | > 7,001.00 | 8 (2.3) |
|  | Missing | 36 (10.5) |
| TYPE OF RESIDENCE | |  |
|  | House | 333 (97.6) |
|  | Apartment | 7 (2.0) |
|  | Farm | 1 (0.2) |
