# Supplementary material for: Cryptic circulation of chikungunya virus in São Jose do Rio Preto, Brazil, 2015–2019
Source: PLoS Negl Trop Dis. 2024 Mar 14;18(3):e0012013. doi: 10.1371/journal.pntd.0012013 (PMC10965090; doi:10.1371/journal.pntd.0012013)
Supplement: S4 Table — (DOCX) [file pntd.0012013.s004.docx]

**S4 Table. Results of seropositivity to CHIKV antibodies, according with anti-CHIKV IgG by ELISA assay and neutralizing antibody titers (PRNT_80_) for the participant paired samples from Vila Toninho during the subcohort study (FB = Baseline 2015/2016, A01 = Follow-up 2016/2017, A02 = Follow-up 2017/2018 and A03 = Follow-up 2018/2019).**

| **ID_Cohort** | **Sample year** | **IgG_CHIKV** | **IgG_MAYV** | **CHIKV_CUT-OFF_80** | **MAYV_CUT-OFF_80** |
| --- | --- | --- | --- | --- | --- |
| 48 | *FB* | Negative | Negative | Negative | Negative |
|  | *A01* | Negative | Negative | Negative | Negative |
|  | *A02* | Negative | Negative | Negative | Negative |
|  | *A03* | Positive | Negative | Negative | Negative |
| 54 | *FB* | Borderline | Negative | Negative | Negative |
|  | *A01* | Borderline | Borderline | Negative | Negative |
|  | *A02* | Positive | Negative | Negative | Negative |
|  | *A03* | Positive | Negative | Positive | Negative |
| 105 | *FB* | Borderline | Negative | Negative | Negative |
|  | *A01* | Negative | Negative | Negative | Negative |
|  | *A02* | Negative | Negative | Negative | Negative |
|  | *A03* | Borderline | Negative | Positive | Negative |
| 307 | *FB* | Positive | Negative | Negative | Negative |
|  | *A01* | Positive | Negative | Negative | Negative |
|  | *A02* | Positive | Borderline | Positive | Negative |
|  | *A03* | Positive | Negative | Positive | Negative |
| 421 | *FB* | Negative | Negative | Negative | Negative |
|  | *A01* | Negative | Negative | Negative | Negative |
|  | *A02* | Borderline | Negative | Negative | Negative |
|  | *A03* | Borderline | Negative | Negative | Negative |
| *506* | *FB* | Negative | Negative | Negative | Negative |
|  | *A01* | Borderline | Negative | Positive | Negative |
|  | *A02* | Borderline | Negative | Negative | Negative |
|  | *A03* | Borderline | Negative | Positive | Negative |
| *537* | *FB* | Negative | Negative | Negative | Negative |
|  | *A01* | Negative | Negative | Negative | Negative |
|  | *A02* | Positive | Negative | Positive | Negative |
|  | *A03* | Positive | Negative | Positive | Negative |
| *560* | *FB* | Negative | Negative | Negative | Negative |
|  | *A01* | Negative | Negative | Negative | Negative |
|  | *A02* | Positive | Negative | Negative | Negative |
|  | *A03* | Positive | Borderline | Positive | Negative |
| 619 | *FB* | Negative | Negative | Negative | Negative |
|  | *A01* | Negative | Negative | Negative | Negative |
|  | *A02* | Negative | Negative | Negative | Negative |
|  | *A03* | Borderline | Negative | Negative | Negative |
| 712 | *FB* | Borderline | Borderline | Negative | Negative |
|  | *A01* | Positive | Positive | Positive | Negative |
|  | *A02* | Positive | Positive | Positive | Negative |
|  | *A03* | Positive | Positive | Positive | Negative |
| 799 | *FB* | Negative | Negative | Negative | Negative |
|  | *A01* | Negative | Negative | Negative | Negative |
|  | *A02* | Negative | Negative | Negative | Negative |
|  | *A03* | Positive | Borderline | Negative | Negative |
| *933* | *FB* | Negative | Negative | Negative | Negative |
|  | *A01* | Negative | Negative | Negative | Negative |
|  | *A02* | Negative | Negative | Negative | Negative |
|  | *A03* | Borderline | Negative | Negative | Negative |
| 961 | *FB* | Negative | Negative | Negative | Negative |
|  | *A01* | Negative | Negative | Negative | Negative |
|  | *A02* | Negative | Negative | Negative | Negative |
|  | *A03* | Positive | Borderline | Negative | Negative |
| 1003 | *FB* | Negative | Negative | Negative | Negative |
|  | *A01* | Negative | Negative | Negative | Negative |
|  | *A02* | Borderline | Negative | Negative | Negative |
|  | *A03* | Borderline | Negative | Negative | Negative |
| 1127 | *FB* | Negative | Negative | Negative | Negative |
|  | *A01* | Negative | Negative | Negative | Negative |
|  | *A02* | Negative | Negative | Negative | Negative |
|  | *A03* | Positive | Negative | Positive | Negative |
| 1517* | *FB* | Negative | Negative | Negative | Negative |

* Sample 1517 was used as a control sample because all years showed negative serological results
