# Supplementary material for: Cryptic circulation of chikungunya virus in São Jose do Rio Preto, Brazil, 2015–2019
Source: PLoS Negl Trop Dis. 2024 Mar 14;18(3):e0012013. doi: 10.1371/journal.pntd.0012013 (PMC10965090; doi:10.1371/journal.pntd.0012013)
Supplement: S5 Table — (DOCX) [file pntd.0012013.s005.docx]

**S5 Table. Association of the presence or absence of symptoms and positive and negative status for CHIKV IgM antibodies determined by ELISA among dengue-suspected patients during the 2019 dengue outbreak.**

| **SYMPTOMS** | | **TOTAL (325; 100.0%)** | | **CHIKV IgM Positive (18; 5.5%)** | | **CHIKV IgM Negative (307; 94.5%)** | | **p Value** | **Adjusted Odds Ratio** | **(95% CI)** |
| --- | --- | --- | --- | --- | --- | --- | --- | --- | --- | --- |
|  |  | **n** | **%** | **n** | **%** | **n** | **%** |  |  |  |
| FEVER | PRESENCE | 239 | 73.5 | 12 | 5.0 | 227 | 95.0 | 0.579 | 0.688 | (0.251-2.046) |
|  | ABSENCE | 86 | 26.5 | 6 | 7.0 | 78 | 90.1 |  |  |  |
| MYALGIA | PRESENCE | 274 | 84.4 | 16 | 5.8 | 254 | 92.7 | 0.747 | 1.635 | (0.415-10.801) |
|  | ABSENCE | 54 | 16.6 | 2 | 3.8 | 52 | 96.3 |  |  |  |
| HEADACHE | PRESENCE | 257 | 79.1 | 14 | 5.4 | 243 | 94.6 | 1.000 | 0.922 | (0.306-3.352) |
|  | ABSENCE | 68 | 20.1 | 4 | 5.9 | 64 | 94.1 |  |  |  |
| EXANTHEMA | PRESENCE | 43 | 13.2 | 5 | 11.6 | 37 | 86.0 | 0.07 | 2.784 | (0.848-8.09) |
|  | ABSENCE | 282 | 86.8 | 13 | 4.6 | 269 | 95.4 |  |  |  |
| VOMITING | PRESENCE | 86 | 26.5 | 9 | 10.5 | 77 | 89.5 | 0,049 | 2.962 | (1.103-7.957) |
|  | ABSENCE | 239 | 73.5 | 9 | 3.8 | 229 | 95.8 |  |  |  |
| NAUSEA | PRESENCE | 172 | 52.9 | 11 | 6.4 | 161 | 93.6 | 0.628 | 1.413 | (0.531-3.96) |
|  | ABSENCE | 153 | 47.1 | 7 | 4.6 | 145 | 4..8 |  |  |  |
| BACKACHE | PRESENCE | 165 | 50.9 | 11 | 6.7 | 154 | 93.3 | 0.468 | 1.589 | (0.597-4.460) |
|  | ABSENCE | 159 | 49.1 | 7 | 4.4 | 152 | 95.6 |  |  |  |
| CONJUNCTIVITIS | PRESENCE | 20 | 6.2 | 2 | 10.0 | 17 | 85.0 | 0.285 | 2.118 | (0.307-8.944) |
|  | ABSENCE | 305 | 93.8 | 16 | 5.24 | 289 | 94.8 |  |  |  |
| PETECHIA | PRESENCE | 33 | 10.2 | 1 | 3.0 | 32 | 96.7 | 1.000 | 0.504 | (0.023-2.936) |
|  | ABSENCE | 292 | 89.8 | 17 | 5.8 | 274 | 93.8 |  |  |  |
| ARTHRITIS | PRESENCE | 77 | 23.7 | 5 | 6.5 | 72 | 93.5 | 0.565 | 1.355 | (0.421-3842) |
|  | ABSENCE | 247 | 76.3 | 13 | 5.3 | 234 | 94.7 |  |  |  |
| ARTHRALGIA | PRESENCE | 64 | 20.0 | 6 | 9.0 | 58 | 91.0 | 0.236 | 2.138 | (0.77-5.93) |
|  | ABSENCE | 260 | 80.0 | 12 | 5.0 | 248 | 95.0 |  |  |  |
| RETROORBITAL PAIN | PRESENCE | 157 | 48.5 | 3 | 1.9 | 150 | 95.5 | 0.043 | 0.284 | (0.062-0.983) |
|  | ABSENCE | 167 | 51.5 | 11 | 6.6 | 156 | 93.4 |  |  |  |
| LEUKOPENIA | PRESENCE | 6 | 1.9 | 1 | 16.7 | 5 | 83.3 | 0.292 | 3.517 | (0.140-27.227) |
|  | ABSENCE | 318 | 98.1 | 17 | 5.3 | 301 | 94.7 |  |  |  |
| TOURNIQUET TEST | PRESENCE | 53 | 16.0 | 2 | 4.0 | 51 | 96.0 | 0.770 | 0.625 | (0.139-2.802) |
|  | ABSENCE | 271 | 84% | 16 | 6% | 255 | 94% |  |  |  |
