# Supplementary material for: Cryptic circulation of chikungunya virus in São Jose do Rio Preto, Brazil, 2015–2019
Source: PLoS Negl Trop Dis. 2024 Mar 14;18(3):e0012013. doi: 10.1371/journal.pntd.0012013 (PMC10965090; doi:10.1371/journal.pntd.0012013)
Supplement: S6 Table — (DOCX) [file pntd.0012013.s006.docx]

**S6 Table. Serological outcome of the anti-CHIKV IgG/IgM (ELISA) and neutralizing antibody titers (PRNT_80_) in samples from dengue-suspected patients in the 2019 outbreak.**

| **ID_Cohort** | **IgM_CHIKV** | **IgG_CHIKV** | **IgG_MAYV** | **CHIKV_CUT-OFF_80** | **MAYV_CUT-OFF_80** |
| --- | --- | --- | --- | --- | --- |
| 6 | Negative | Positive | Negative | Positive | Negative |
| 8 | Negative | Positive | Negative | Positive | Negative |
| 11 | Negative | Positive | Negative | Positive | Negative |
| 18 | Negative | Positive | Negative | Positive | Negative |
| 70 | Negative | Borderline | Negative | Negative | Negative |
| 71 | Negative | Borderline | Negative | Negative | Negative |
| 72 | Negative | Positive | Negative | Negative | Negative |
| 82 | Negative | Positive | Negative | Negative | Negative |
| 87 | Positive | Positive | Negative | Positive | Negative |
| 88 | Negative | Borderline | Negative | Positive | Negative |
| 94 | Negative | Positive | Negative | Positive | Negative |
| 111 | Positive | Negative | Negative | Positive | Negative |
| 563 | Positive | Negative | Negative | Negative | Negative |
| 584 | Negative | Borderline | Negative | Negative | Negative |
| 683 | Negative | Borderline | Negative | Positive | Negative |
| 743 | Negative | Positive | Negative | Positive | Negative |
| 803 | Negative | Borderline | Negative | Negative | Negative |
| 1147 | Negative | Positive | Negative | Positive | Negative |
| 1279 | Negative | Positive | Negative | Positive | Negative |
| 1332 | Borderline | Negative | Negative | Negative | Negative |
| 1339 | Positive | Negative | Negative | Positive | Negative |
| 1470 | Negative | Positive | Negative | Positive | Negative |
| 1485 | Negative | Positive | Negative | Negative | Negative |
| 1483 | Negative | Positive | Negative | Positive | Negative |
| 1493 | Negative | Positive | Negative | Positive | Negative |
| 1496 | Negative | Positive | Negative | Positive | Negative |
| 1497 | Negative | Positive | Negative | Positive | Negative |
| 1505 | Negative | Positive | Negative | Positive | Negative |
| 1506 | Negative | Positive | Negative | Positive | Negative |
| 1638 | Positive | Negative | Negative | Positive | Negative |
| 1676 | Negative | Positive | Negative | Positive | Negative |
| 1731 | Positive | Negative | Negative | Positive | Negative |
| 1871 | Negative | Positive | Negative | Positive | Negative |
| 1972 | Negative | Positive | Negative | Positive | Negative |
| 2000 | Negative | Borderline | Negative | Positive | Negative |
| 2037 | Negative | Positive | Positive | Positive | Positive |
| 2039 | Negative | Positive | Negative | Positive | Negative |
| 2077 | Negative | Positive | Negative | Positive | Negative |
| 2102 | Negative | Positive | Negative | Positive | Negative |
| 2105 | Positive | Borderline | Negative | Positive | Negative |
| 2128 | Negative | Positive | Positive | Positive | Positive |
| 2196 | Borderline | Negative | Negative | Positive | Negative |
| 2180 | Negative | Negative | Negative | Negative | Negative |
| 2202 | Negative | Positive | Negative | Positive | Negative |
| 2411 | Positive | Negative | Negative | Positive | Negative |
| 2256 | Borderline | Negative | Negative | Positive | Negative |
| 2543 | Negative | Borderline | Negative | Positive | Negative |
| 2729 | Positive | Negative | Positive | Positive | Positive |
| 3045 | Negative | Positive | Negative | Positive | Negative |
| 3103 | Positive | Negative | Negative | Positive | Negative |
| 3415 | Negative | Positive | Negative | Positive | Negative |
| 3983 | Negative | Borderline | Negative | Positive | Negative |
| 3992 | Borderline | Negative | Negative | Positive | Negative |
| 4041 | Positive | Borderline | Borderline | Positive | Negative |
| 4307 | Positive | Negative | Negative | Positive | Negative |
| 4629 | Negative | Positive | Negative | Positive | Negative |
| 4637 | Negative | Positive | Negative | Positive | Negative |
| 4638 | Negative | Positive | Negative | Negative | Negative |
| 4676 | Negative | Positive | Negative | Positive | Negative |
| 5091 | Negative | Borderline | Negative | Negative | Negative |
| 5120 | Negative | Positive | Negative | Negative | Negative |
| 5177 | Negative | Positive | Negative | Positive | Negative |
| 5482 | Negative | Positive | Negative | Positive | Negative |
| 5658 | Positive | Negative | Negative | Positive | Negative |
| 5755 | Negative | Positive | Negative | Positive | Negative |
| 6485 | Positive | Negative | Negative | Positive | Negative |
