# Supplementary material for: Cryptic circulation of chikungunya virus in São Jose do Rio Preto, Brazil, 2015–2019
Source: PLoS Negl Trop Dis. 2024 Mar 14;18(3):e0012013. doi: 10.1371/journal.pntd.0012013 (PMC10965090; doi:10.1371/journal.pntd.0012013)
Supplement: S7 Table — (DOCX) [file pntd.0012013.s007.docx]

**S7 Table The overdilution samples from dengue-suspected patients in the 2019 outbreak, which supported CHIKV and MAYV cross-reaction during neutralizing antibody titration (PRNT_80_).**

| Dilution | ID_VE | IgM_CHIKV | IgG_CHIKV | IgG_MAYV | **CHIKV_CUT-OFF_80** | **MAYV_CUT-OFF_80** |
| --- | --- | --- | --- | --- | --- | --- |
| 1:20 | 2037 | Negative | Positive | Positive | Negative | Positive |
|  | 2128 | Negative | Positive | Positive | Positive | Positive |
|  | 2729 | Positive | Negative | Negative | Positive | Negative |
| 1:40 | 2037 | Negative | Positive | Positive | Negative | Positive |
|  | 2128 | Negative | Positive | Positive | Positive | Positive |
|  | 2729 | Positive | Negative | Negative | Positive | Negative |
| 1:80 | 2037 | Negative | Positive | Positive | Negative | Positive |
|  | 2128 | Negative | Positive | Positive | Negative | Positive |
|  | 2729 | Positive | Negative | Negative | Positive | Negative |
| 1:160 | 2037 | Negative | Positive | Positive | Negative | Positive |
|  | 2128 | Negative | Positive | Positive | Negative | Negative |
|  | 2729 | Positive | Negative | Negative | Positive | Negative |
| 1:320 | 2037 | Negative | Positive | Positive | Negative | Positive |
|  | 2128 | Negative | Positive | Positive | Negative | Negative |
|  | 2729 | Positive | Negative | Negative | Positive | Negative |
| 1:640 | 2037 | Negative | Positive | Positive | Negative | Negative |
|  | 2128 | Negative | Positive | Positive | Negative | Negative |
|  | 2729 | Positive | Negative | Negative | Negative | Negative |
| 1:1280 | 2037 | Negative | Positive | Positive | Negative | Negative |
|  | 2128 | Negative | Positive | Positive | Negative | Negative |
|  | 2729 | Positive | Negative | Negative | Negative | Negative |
